# Heterolytic Splitting of Molecular Hydrogen by Frustrated and Classical Lewis Pairs: A Unified Reactivity Concept

**DOI:** 10.1038/s41598-017-16244-1

**Published:** 2017-11-22

**Authors:** Gabriella Skara, Freija De Vleeschouwer, Paul Geerlings, Frank De Proft, Balazs Pinter

**Affiliations:** Quantum Chemistry Group, Member of the QCMM VUB-UGent Alliance Research Group, Vrije Universiteit Brussel (VUB), Pleinlaan 2, B-1050 Brussels, Belgium

## Abstract

Using a set of state-of-the-art quantum chemical techniques we scrutinized the characteristically different reactivity of frustrated and classical Lewis pairs towards molecular hydrogen. The mechanisms and reaction profiles computed for the H_2_ splitting reaction of various Lewis pairs are in good agreement with the experimentally observed feasibility of H_2_ activation. More importantly, the analysis of activation parameters unambiguously revealed the existence of two reaction pathways through a low-energy and a high-energy transition state. An exhaustive scrutiny of these transition states, including their stability, geometry and electronic structure, reflects that the electronic rearrangement in low-energy transition states is fundamentally different from that of high-energy transition states. Our findings reveal that the widespread consensus mechanism of H_2_ splitting characterizes activation processes corresponding to high-energy transition states and, accordingly, is not operative for H_2_-activating systems. One of the criteria of H_2_-activation, actually, is the availability of a low-energy transition state that represents a different H_2_ splitting mechanism, in which the electrostatic field generated in the cavity of Lewis pair plays a critical role: to induce a strong polarization of H_2_ that facilities an efficient end-on acid-H_2_ interaction and to stabilize the charge separated “H^+^–H^−^” moiety in the transition state.

## Introduction

**Figure 1:**
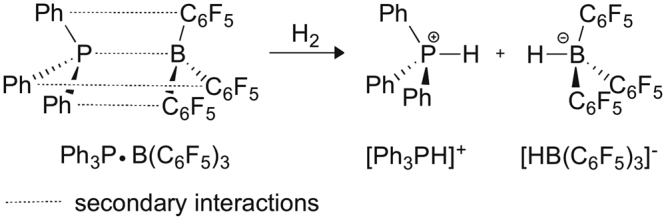
A representative frustrated Lewis pair (FLP) and direct products of heterolytic H_2_-splitting.

The activation of molecular hydrogen has been an active field of research for over decades. The discovery of frustrated Lewis pairs (FLPs), a metal free catalyst that is capable of heterolytically cleaving H_2_ under mild conditions has been in the focus of research since 2007. Since then, a vast number of synthetic, mechanistic and theoretical works appeared in the literature applying FLPs to activating H_2_ as well as other small molecules. In 2006 Stephan and coworkers reported the first non-classical phosphonium-borate compound, ^+^p-(Mes_2_PH)–C_6_F_4_–(BH(C_6_F_5_)_2_)^−^, that can reversibly liberate H_2_ and yields p-(Mes_2_P)C_6_F_4_(B(C_6_F_5_)_2_ at temperatures above 100 °C, whereas H_2_ uptake by the latter can take place even at 25 °C to regenerate the original salt^1^. The term, “frustrated Lewis pair” (FLP) was introduced in 2007^2^ describing the above mentioned non-classical Lewis pair compounds that are “associations” of a Lewis acid and a Lewis base that are hindered by steric and/or electronic factors from forming strong, datively bound classical Lewis adducts (Fig. 1). Steric congestion prevents the frontier orbitals of the acid and base to ideally overlap and to form a dative bond; however, non-covalent interactions between the bulky substituents and functional groups on the acid and base stabilize the adduct of reactants, called frustrated/encounter complex.

Since the articulation of the notion, a vast number of studies probing the chemistry of FLPs and their applications for various transformations have emerged and the developments made in less than a decade are already too diverse and numerous to list comprehensively. Very recent articles and reviews of Stephan^3–5^ summarize and discuss the major developments, areas of advancement, applications and understanding of the reactivity that have been made in the field of FLP chemistry.

The studied reactions of FLPs were systematically extended revealing that various phosphonium-borate compounds not only release and bind H_2_ reversibly^6^, but also act as hydrogenation catalysts for imines, protected nitriles and aziridines^7^, and serve as a hydride source for the stoichiometric reduction of aldehydes^8^. A series of different donors and acceptors have been investigated rapidly^9–21^ and different mechanistic scenarios have been proposed and scrutinized experimentally^9,10,22–25^ as well as by quantum chemical calculations^12,18,22,26–37^. Both intermolecular and intramolecular (linked)^22,26,33,34^ systems have been studied, and the choice of polar substrates for catalytic hydrogenations was also extended to, for example, silyl enol ethers and enamines^9,11,12,22,26,32,38^. On the base side, a variety of phosphine derivatives, i.e. ferrocenyl phosphines^13,14^, imine^11,15,16^, amine^9,12,15^, pyridine^10^ and carbene^15,18–21^ compounds have been reported, while the acid side has been mostly limited to borane derivatives^16,39–42^ so far. For intramolecular systems, the fragments that tether the acid and base centers most often include p–C_6_F_4_, methyphenyl^22^ and ethylene^26,33^ moieties and a directly linked phospanylborane system^34^ is also known.

One of the most studied reactions of FLPs is the direct catalytic hydrogenation of organic substrates with polar double bonds. Also, the reaction of FLPs with a variety of other small molecules, such as olefins^43–46^, alkynes^47^, polyaromatic system, i.e. anthracene^48,49^, sterically hindered anilines^50^, pyridines^51^, quinolones^52^ and a range of element oxides^53^ have been studied. In addition, the catalytic hydroamination^54^ and CO_2_ reduction^55^ and applications to polymerization^56–59^ have also been investigated and developed.

Theoretical investigations appeared soon^9,10,12,18,19,22,26–33,35,36^ in order to understand the mechanistic details of the related reactions. Despite the extensive and thorough early studies in the field, the actual mechanism of hydrogen activation by FLPs has become a matter of controversy^31,37,47,60–69^. The first computational study on the mechanism appeared in 2008 authored by Rokob *et al*.^27^, in which a combination of DFT and *ab initio* methods was used to gain detailed insight in the mechanism of the *t*Bu_3_P + B(C_6_F_5_)_3_ + H_2_ system; the reaction that later became the paradigm case of FLPs. On the basis of their computational analysis it was concluded that the formerly intuitively proposed side-on and end-on approaches of H_2_ either to B(C_6_F_5_)_3_ or *t*Bu_3_P were unfavorable due to Pauli repulsion between interacting fragments. The delocalization of π electrons of the aryl groups to the empty p orbital of boron was found to limit the effect of stabilizing σ donation from H_2_ to boron precluding a direct R_3_B ^…^H_2_–type complexation. It was also pointed out that the rather facile hydrogen-splitting reaction could hardly be explained in terms of a termolecular collision of the reactants, and thus a weak pre-association of the acid and base molecules was envisioned and identified on the PES as a key ingredient of the reaction. This preorganized donor-acceptor complex, was shown to be a highly flexible species, held together by weak, secondary, non-covalent interactions, including multiple C–H^…^F interactions and dispersion.

**Figure 2:**
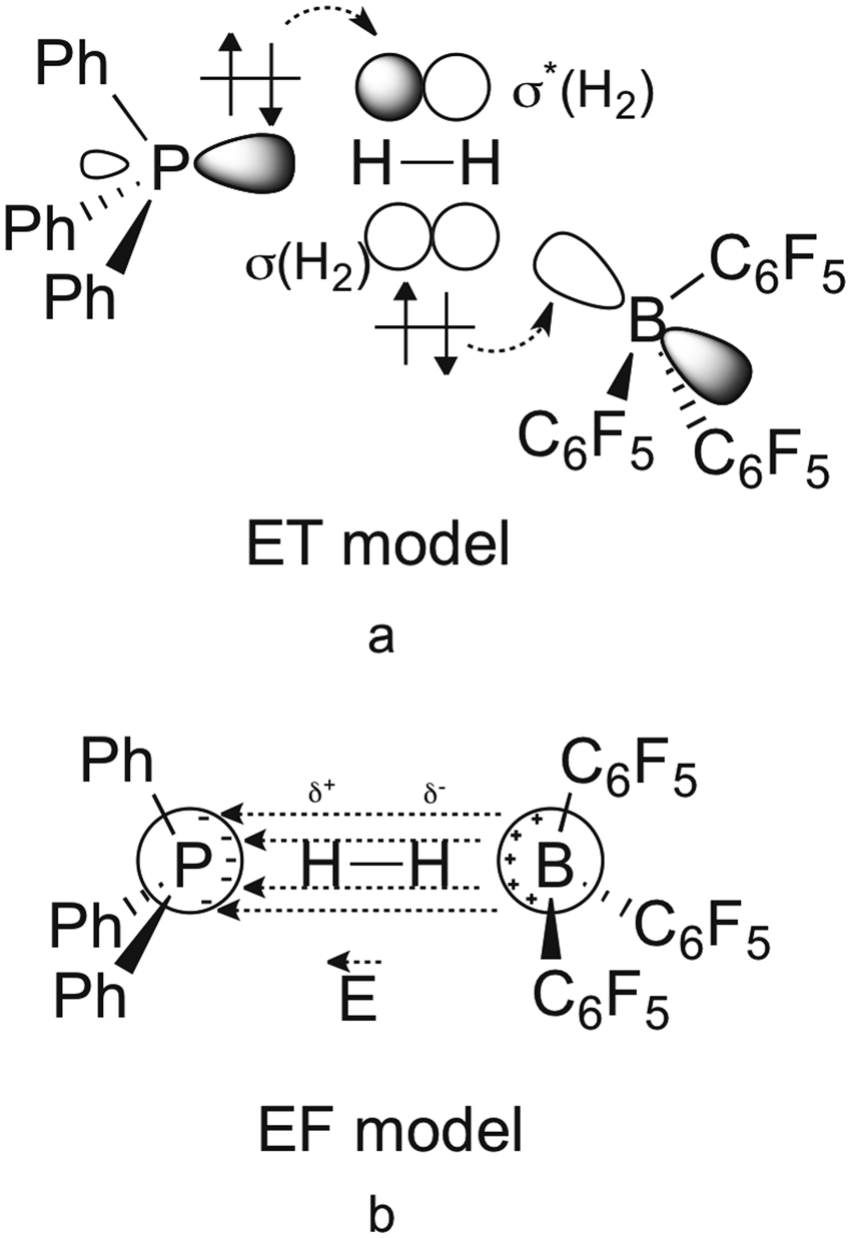
Schematic views of the proposed electron transfer (ET) (**a**) and electric field (EF) (**b**) based interpretations of H_2_ activation.

While it is not scrutinized in much detail in earlier studies, it is generally accepted that the flexibility of FLPs allows the H_2_ molecule to easily enter the “reactive pocket” of the system^27,60,70^. The energy requirement of this process might be estimated from the energy demand of 6.1 kcal mol^−1^ calculated for a representative intramolecular FLP system to adopt an open gauche conformer. As a next step, a single, low-lying *early* transition state (TS) from the reactants to the products was identified, in which the H_2_ is close to the base and acid reactive centers and interacts with them simultaneously. From these results a generalized reactivity model was proposed, in which the mechanism proceeds by a simultaneous electron-transfer (ET) occurring from the lone pair of (*t*Bu)_3_P to the antibonding (σ*) orbital of H_2_ and a donation from the σ bonding orbital of H_2_ to the Lewis acid, B(C_6_F_5_)_3_ in a push-pull manner (Fig. 2a). This facilitates a progressive weakening of the H–H bond along the reaction pathway and ultimately leads to the heterolytic cleavage of H_2_. The structurally flexible encounter complex provides a range of optimal preorganized active centers, nevertheless, it is only present in low-concentration due to the unfavorable entropy of association.

Opposed to the electron transfer (ET) model of Rokob described above, Grimme and co-workers proposed an alternative, conceptually distinct reactivity model to interpret the facile heterolytic cleavage^60^. As depicted in Fig. 2b, the key concept of the EF model is that the cleavage takes place as a result of polarization of H_2_ by the strong homogeneous electric field (EF) present in a reactive pocket created by the active centers of the FLPs. Accordingly, within the framework of the EF model, the rate-determining step of H_2_ cleavage is the entrance of the essentially intact H_2_ into to the interior of the FLP, which was suggested to be hindered only by steric repulsion and the unfavorable deformation of the complex. The similar chemical behavior of different FLPs was proposed to originate from similarity of the electric field characteristics.

Based on both theoretical and experimental studies it was early agreed upon that secondary interactions play a prominent role in the mechanism, not only in the reactant state but also along the entire process including the transition state^9,27,60^. Also, the existence of a preorganized entity is now univocally accepted. According to simulations using an explicit solvation model, the encounter complex can be present in solvent in a small, but relevant concentration and accordingly, the reaction takes place by the intermediacy of this reactive species. Finally, recently the association of Mes_3_P and B(C_6_F_5_)_3_ into transient species (K = 0.5 M^−1^, ΔG^0^ (298 K) = 0.4 kcal mol^−1^) was confirmed experimentally in a milestone study by Rocchigiani *et al*. using ^19^F, ^1^H HOESY, diffusion and temperature-dependent ^19^F and ^1^H NMR techniques^68^.

In an attempt to settle the debate on the H_2_ cleavage mechanism, Camaioni *et al*. presented a detailed analysis of the reaction of a *classical* Lewis acid-base pair (CLP), NH_3_–BCl_3_ with H_2_, by using the Localized Molecular Orbital Energy Decomposition Analysis (LMOEDA)^62^. Although the selected CLP does not split H_2_, the applied electronic structure theories could be used to explore the PES in the region relevant to H_2_ activation in FLPs. They revealed that the dominant stabilizing factor at the TS is a charge transfer interaction, and that the electric field clearly plays a role in the polarization of H_2_, but its contribution to the overall interaction energy is small compared to the orbital interactions.

In 2013, Rokob and Pápai and co-workers revisited and reassessed the applicability of the two mechanistic views by examining the reactions of a representative set of six FLPs with H_2_ that have been characterized experimentally^31^. It was found that in the TSs, the base^…^ H–H^…^ acid fragment is actually not linear but has a characteristic bent arrangement, with a general tendency for end-on base^…^ H_2_ and side-on acid^…^ H_2_ interactions. Plotting the electric field in the studied TSs revealed that the fields are actually extremely inhomogeneous, and that there is no “cavity” region with a field strength that is sufficient to reduce the H–H activation barrier, i.e. a field strength of ~0.09 a.u. or larger. Actually, in TSs with relatively large base^…^acid distance the electric field (EF) strength in the interior is about 0.02–0.04 a.u. whereas the direction of the EF vector does not point in the direction of the H–H bond. Considering only the components parallel to the H–H axes in the TSs, the sign of the EF is appropriate to polarize H_2_ in the observed direction, but its magnitude is below the critical value of ~0.08 a.u., still preventing an explanation of the cleavage in terms of the barrier height or H–H distance. Thus, it was concluded that the EF generated by the FLP is not sufficient to account for the observed hydrogen splitting and that the EF model does not provide an explanation for the observed bent geometry of transition states either.

In a recent contribution^71^ we scrutinized the contribution of many different types of weak interactions to the formation of FLPs by deploying an arsenal of state-of-the-art computational techniques including the Non-Covalent Interactions (NCI) method^72,73^, Quantum Theory of Atoms in Molecules, (QTAIM)^74^ and a Ziegler-Rauk energy decomposition analysis^75,76^ coupled to the Natural Orbital for Chemical Valence (NOCV) analysis^77–80^. The importance of dispersion, π–π stacking and C–F^…^H bonds in the formation of FLPs has been repeatedly anticipated in earlier studies, for which these computational techniques could provide quantitative evidence. Moreover, interaction energy decomposition and NOCV analyses clearly supported the earlier proposed lack of dative bond between donor and acceptor centers in FLPs.

In this study we focus on the reactivity of representative FLPs and CLPs towards molecular hydrogen using again a complementing set of *in silico* instruments to answer the simple but critical question that, as we believe, arises from the findings of earlier studies. Namely; *do CLPs and FLPs* ‘*react*’ *with H*_2_ *in the same way?* According to the consensus understanding of the reactivity of frustrated Lewis pairs towards H_2_, FLPs and CLPs do actually interact, from electronic aspects, with H_2_ alike; classical Lewis-pairs fulfill all electronic prerequisites of H_2_ splitting, including the ability of electron donation to the σ*(H_2_) orbital and withdrawal from σ(H_2_) augmented by a weak electrostatic field. Still, FLPs and CLPs behave very differently in practice, and even thermodynamically unstable CLPs do not react with H_2_. To the best of our knowledge this controversy has not been addressed before.

The quenching of the acid and base reactivities in CLPs via dative bond formation is a conceptually compelling explanation for the unreactive nature of CLPs. The energy-related aspects of this argument are, however rather hand-waving than trivial: the splitting of H_2_ by FLPs proceeds without a notable barrier (~5 kcal mol^−1^) whereas CLPs has a relative stability of about −10 to −30 kcal mol^−1^ to free reactants^81^. Accordingly, if H_2_ activation indeed takes place through a similar transition state for CLPs and FLPs, i.e. the same way for the two types of systems, many CLPs should activate H_2_ (at least from a kinetic point of view), in extreme cases passing through an activation barrier as low as 15 kcal mol^−1^. And, yet, only one CLP, lut–B(C_6_F_5_)_3_^10,37^, which was recently termed as a “hidden FLP”^3^, activates H_2_. In this study we aim at shedding light on whether there are actually two conceptually different H_2_-activation mechanisms corresponding to activating and non-activating systems or whether a phenomenon called “steric acceleration” (that takes also into account reactivity quenching effects) indeed differentiates FLPs from CLPs. Steric acceleration, in general, embodies two activation barrier lowering effects of bulky substituents: destabilization of the reactant state through steric repulsions between large groups and stabilization of the transition state by secondary interactions between these bulky substituents. The determining effect of steric acceleration of bulky ligands on the rate of, for example, Suzuki-Miyaura cross-coupling reactions has been recently demonstrated by Szilvási and Veszprémi^82^.

## Results and Discussion

**Figure 3:**
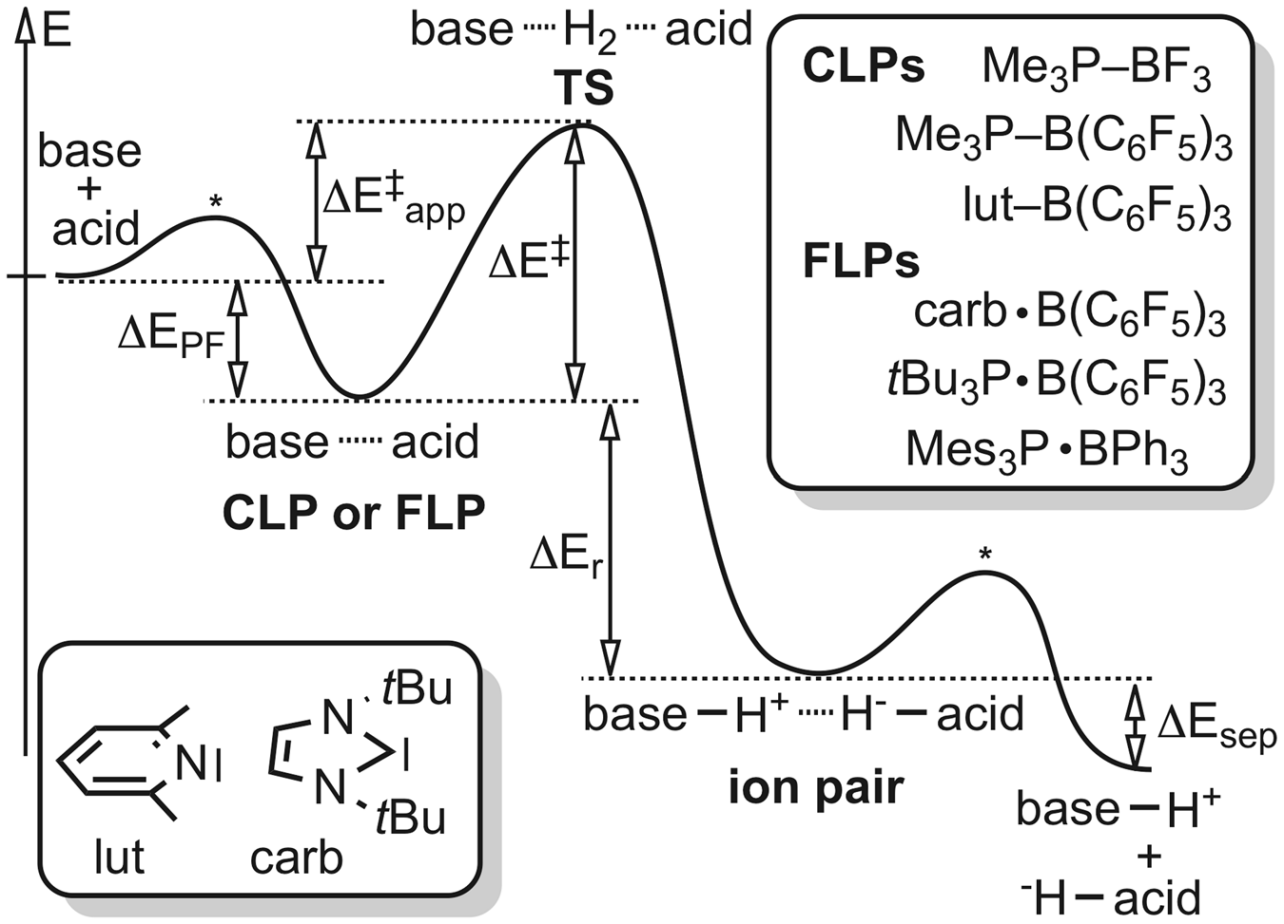
Definition of stationary points, activation- and reaction parameters on a general reaction profile for the heterolytic cleavage of H_2_ by FLPs and CLPs, given together with the investigated systems (top right) and structure of lut and carb (bottom left). CLPs and FLPs are systematically referred to as base–acid and base · acid, respectively, in this study.

In this comprehensive computational study we systematically investigated the reaction of six Lewis pairs with molecular hydrogen, shown schematically in Fig. 3, including three CLPs (Me_3_P–BF_3_, Me_3_P–B(C_6_F_5_)_3_ and lut–B(C_6_F_5_)_3_), amongst which only lut–B(C_6_F_5_)_3_ facilitates the splitting of H_2_, and three FLPs (carb · B(C_6_F_5_)_3_, *t*Bu_3_P · B(C_6_F_5_)_3_, Mes_3_P · BPh_3_), amongst which only Mes_3_P · BPh_3_ does not promote heterolytic H_2_ splitting.

Accordingly, these systems cover all possible types of scenarios, as well as they span a representative chemical space allowing us to investigate the effect of the donor atom and substituents of the Lewis base. Most importantly, through these systems we can unambiguously demonstrate the operation of two distinct mechanisms of activation of H_2_ by Lewis-pairs, which have clear manifestations in the energy-related activation parameters and in the geometry and electronic structure of transition states. Figure 3 defines the most relevant activation and reaction parameters including pair formation energy of FLPs and CLPs (ΔE_PF_), activation energy (ΔE^‡^), apparent barrier (ΔE^‡^_app_), reaction energy (ΔE_r_) and ion separation energy (ΔE_sep_), whereas the corresponding computed solution-state Gibbs free energy and electronic energy values are listed in Table 1.

**Table 1:** Pair formation energy (ΔE_PF_), apparent barrier (ΔE^‡^_app_), activation energy (ΔE^‡^), reaction energy (ΔE_r_), and ion separation energy (ΔE_sep_) and their respective Gibbs free energy values in solution (benzene): ΔG_PF_, ΔG^‡^_app_, ΔG^‡^, ΔG_r_, ΔG_sep_. All values are in kcal mol^−1^.

	ΔE_PF_	ΔE^‡^_app_	ΔE^‡^	ΔE_r_	ΔE_sep_	ΔG_PF_	ΔG^‡^_app_	ΔG^‡^	ΔG_r_	ΔG_sep_
Me_3_P–BF_3_	−14.6	14.0	28.6	20.7	92.8	−7.6	32.6	40.2	28.6	22.4
Me_3_P–B(C_6_F_5_)_3_	−28.6	0.1	28.7	11.8	73.7	−11.4	24.9	36.3	16.0	16.1
lut–B(C_6_F_5_)_3_	−12.8	−2.9	9.9	−13.6	77.0	7.6	24.0	16.3	−7.3	18.8
carb · B(C_6_F_5_)_3_	−8.8	−8.5	0.3	−50.4	70.9	9.5	23.2	13.7	−37.0	13.1
*t*Bu_3_P · B(C_6_F_5_)_3_	−10.4	−4.3	6.1	−19.7	69.1	4.8	22.3	17.4	−10.2	13.8
Mes_3_P · BPh_3_	−14.1	7.9	22.0	12.8	73.4	4.6	32.7	28.1	19.9	9.4

The computed solution-state ΔG_r_ and activation ΔG^‡^ parameters are in good agreement with the available experimental findings: heterolytic H_2_ splitting is not preferred for Me_3_P–BF_3_, Me_3_P–B(C_6_F_5_)_3_ and Mes_3_P · BPh_3_ whereas it takes place through a thermally accessible activation barrier and is an exothermic process in the case of lut–B(C_6_F_5_)_3_, carb · B(C_6_F_5_)_3_ and *t*Bu_3_P · B(C_6_F_5_)_3_. Also, these critical differences between non-activating and activating systems, respectively, are clearly apparent in the corresponding electronic energy originated parameters, ΔE^‡^ and ΔE_r_, allowing us to interpret the most critical differences between systems with the evolution of the underlying electronic structures along the reaction path. Most essentially, the ΔE^‡^ values for Me_3_P–BF_3_ and Me_3_P–B(C_6_F_5_)_3_ indicate kinetically forbidden reactions (ΔE^‡^~28 kcal mol^−1^) whereas for H_2_-activating systems (lut–B(C_6_F_5_)_3_, carb · B(C_6_F_5_)_3_ and *t*Bu_3_ · B(C_6_F_5_)_3_) the computed ΔE^‡^ values of 0–10 kcal mol^−1^ imply an easy H_2_ activation route. For Mes_3_P · BPh_3_, the activation energy of 22.0 kcal mol^−1^ is already high and together with the loss of entropy when going from the Lewis pair to the transition state it results in a prohibiting activation Gibbs free energy barrier (ΔG^‡^_app_ = 32.7 kcal mol^−1^) and accordingly, no H_2_-splitting has been documented for this system.

**Figure 4:**
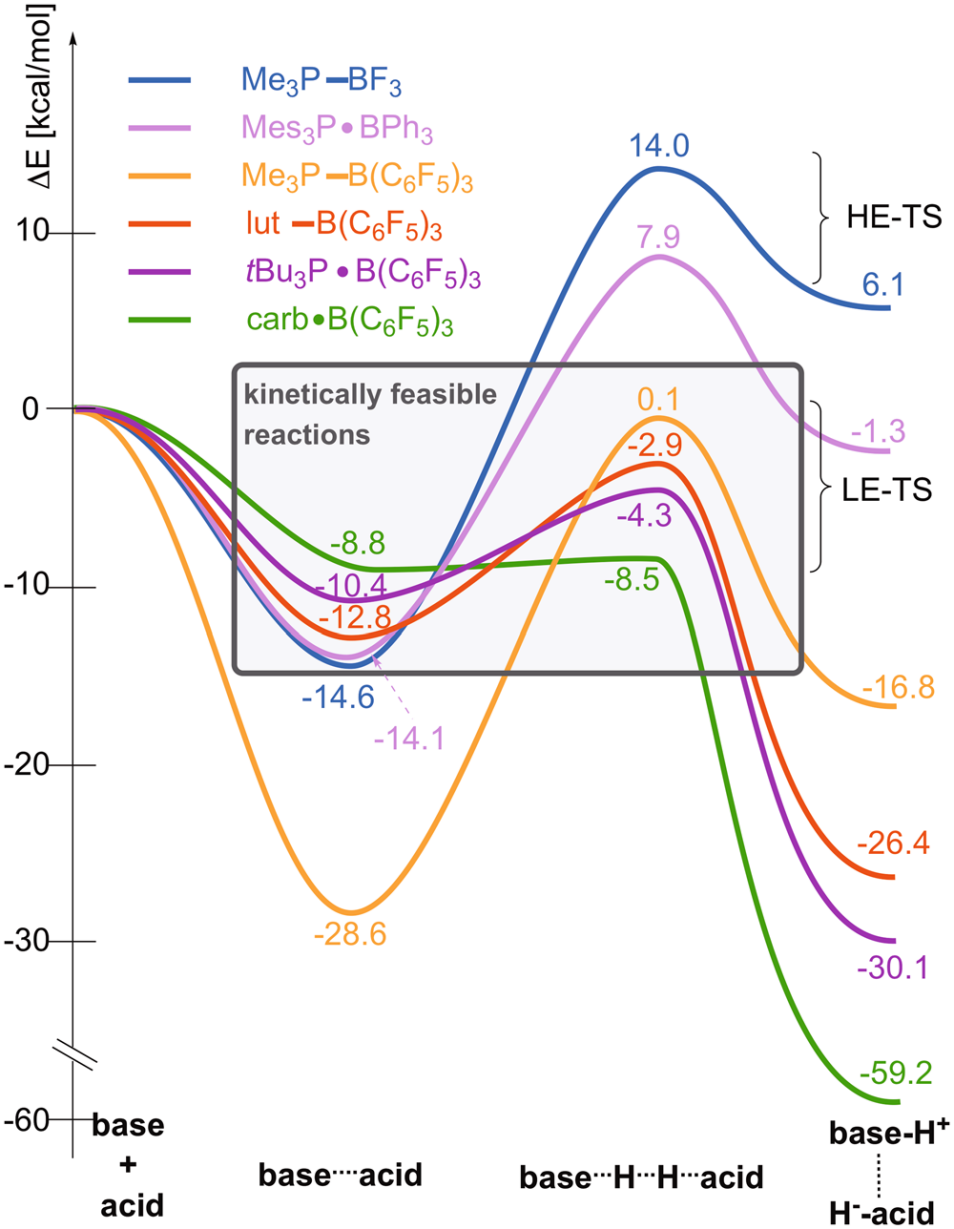
Computed energy profiles (ΔE) of the investigated reactions serving as a basis for the concept of low-energy and high-energy transition states (LE-TS and HE-TS). FLPs and CLPs are not defined based on their relative stability but based on the lack or presence of a dative bond between the acid and base, respectively.

With Fig. 4, which illustrates the computed energy (ΔE) profiles, we aim to give a sound basis for an intuitive conceptual understanding and a unified reactivity concept for the H_2_ activation of Lewis-pairs. First, as Fig. 4 clearly shows, two types of Lewis-pairs and two types of transition states, low-energy (LE-TS) and high-energy TSs (HE-TS), can be distinguished.

According to this energy-based classification, we can unambiguously state that the high-energy transition state of Me_3_P–BF_3_ and Mes_3_P · BPh_3_ is characteristically different from the low-energy transition states revealed for the other systems. It is also clear from Fig. 4 that H_2_ splitting is kinetically feasible for systems that traverse through a low-energy transition state and a non-stable Lewis pair (ΔE_PF_ = −5–−15 kcal mol^−1^), which is most often an FLP. The quenching of acid and base reactivity, an appealing electronic structure-related concept for accounting for the reactivity difference of FLPs and CLPs, can be intuitively witnessed in the significantly more negative relative energy of stable Lewis pairs, such as Me_3_P–B(C_6_F_5_)_3_ (Fig. 4). In these cases the high stability of the Lewis pair inevitably manifests in a prohibiting activation barrier for H_2_ splitting, which is 28.7 kcal mol^−1^ for Me_3_P–B(C_6_F_5_)_3_.

There are CLPs, however that activate H_2_, such as lut–B(C_6_F_5_)_3_, in spite of the dative bond linking the acid and the base, i.e. reactants with quenched reactivity. Instead of terming these systems as “hidden FLPs” for their behaviour, it is more important to realize that these are not so stable classical Lewis pairs with a relative stability of about −10 kcal mol^−1^ to free reactants (e.g. lut–B(C_6_F_5_)_3_ in Fig. 4), held together by a weak dative bond. Accordingly, these CLPs are also capable of activating H_2_, if they exhibit a low-energy transition state. Classical Lewis pair Me_3_P–BF_3_, with ΔE_PF_ = −10 kcal mol^−1^, on the contrary, showcases the scenario when the large activation barrier of H_2_ splitting originates from the high energy of the corresponding transition state and not from the stability of the Lewis pair.

The ΔE^‡^_app_ values, which give the relative energy of transition states to separated reactants, i.e. ΔE^‡^_app_ eliminates all energy-related manifestations of reactivity quenching and, thus, puts all systems on equal footing, are considerably positive (8–14 kcal mol^−1^) for Lewis pairs with HE-TS whereas they vary about −5 kcal mol^−1^ for H_2_-activating systems with LE-TS. This simple observation strongly implies the operation of two different activation mechanisms through the two types of transition states. Moreover, while a large ΔE^‡^_app_ value (e.g. in Me_3_P–BF_3_) is in agreement with the key aspects of the consensus hydrogen activation mechanism that includes a significant electron donatation to the high-lying σ* orbital of H_2_, the barrierless splitting for H_2_-activating systems with LE-TSs does not conform with such a very energy demanding base → σ*(H_2_) donation process.

Table 2 lists the most salient structural features of the optimized transition states, such as the H–H, base^…^H, acid^…^H and base^…^acid interatomic distances and base–H–H and H–H–acid angles. It is critical to realize that a minor donation from the base to the antibonding σ* of H_2_ is expected to trigger a prominent elongation of the H–H bond, as this orbital has a nodal plane between the two hydrogen centers. In addition, electron withdrawing from the bonding σ(H_2_) orbital by the base would further lengthen the H–H bond to some extent. In line with these notions, a significantly stretched H–H bond of about 0.95–0.98 Å is revealed for the transition states of the non-activating Me_3_P–BF_3_ and Mes_3_P–BPh_3_ implying that these base/acid-H_2_ interactions are indeed in operation in high-energy TSs. In low-energy transition states (LE-TSs), in contrast, the H–H bonds are barely longer, fluctuating about 0.8 Å (Table 2), than the equilibrium distance in free H_2_ (0.76 Å). This is a controversial observation in the sense that it shows clearly that the simplest manifestation of base → σ*(H_2_) donation is actually not apparent in the transition states of H_2_-activating systems with LE-TSs. In addition, the computed base^…^H distances are characteristically longer (by about 0.4–0.6 Å) for LE-TSs than for HE-TSs (e.g. 2.39 Å in Me_3_P–B(C_6_F_5_)_3_ *vs*. 1.75 Å in Me_3_P–BF_3_) further questioning the importance of electron donation from the base to σ*(H_2_) in low-energy transition states.

**Table 2:** Most characteristic structural metrics of transition states given in Å and in degree.

	d_H–H_	d_acid_…_H_	d_base_…_H_	d_base_…_acid_	< _acid-H-H_	< _H-H-base_
Me_3_P–BF_3_	0.98	1.40	1.75	3.24	102.7	172.1
Me_3_P–B(C_6_F_5_)_3_	0.79	1.70	2.39	4.08	100.1	165.5
lut–B(C_6_F_5_)_3_	0.79	1.74	1.94	3.82	108.2	163.5
carb · B(C_6_F_5_)_3_	0.81	1.90	1.96	4.27	135.7	174.8
*t*Bu_3_P · B(C_6_F_5_)_3_	0.79	1.81	2.33	4.47	115.9	161.3
Mes_3_P · BPh_3_	0.95	1.40	1.77	3.93	134.2	156.1

Other interesting features are the computed H–H–B angles for these LE-TSs, which vary between 110° and 135°, instead of being about 90° as it would be expected for an acid side-on approach to H_2_. In fact, these H–H–B angles are more consistent with an end-on interaction of the acid with H_2_ for these structures rather than with a side-on electron withdrawing process that was proposed earlier. These striking structural differences between transition states further support our notion of two distinct H_2_-activation mechanisms for systems with low-energy TSs and high-energy TSs.

Earlier theoretical studies admittedly recognized these striking structural differences between transition states and classified them as “early” and “late” referring to their relative position on the reaction coordinate. As the H–H distance is the main component of the reaction coordinate, it is natural terming transition states with short H–H distance as “early” whereas with significantly elongated H–H distance as “late”. To our best knowledge, however, this geometry-based distinction of TSs has not been linked to an electronic structure (and energy) differentiation of transition states, however, it is crucial in understanding the feasibility of H_2_ splitting by Lewis pairs. As a matter of fact, as the terms “early” and “late” is used for these systems, we suspect a widespread anecdotal view in this field that the activation process of H_2_ through early and late TSs is somehow the same; the same sort of activation takes place “earlier” for one system whereas “later” for another. In this context, it is critical to realize, however, that these transition states represent the highest-energy structure along the corresponding H–H splitting process and, accordingly the most critical electronic rearrangements are taking place at these specific geometries. If the geometries are critically different then the electronic structure rearrangements must be different as well. In other words, systems with early TSs do not pass a reaction coordinate that is characteristic for late TSs, and vice versa. In the following analyses we convincingly demonstrate that low-energy transition states (geometrically early) and high-energy transitions states (geometrically late) differ in their electronic structure and, correspondingly, they represent two fundamentally different H_2_-activation mechanisms.

**Figure 5:**
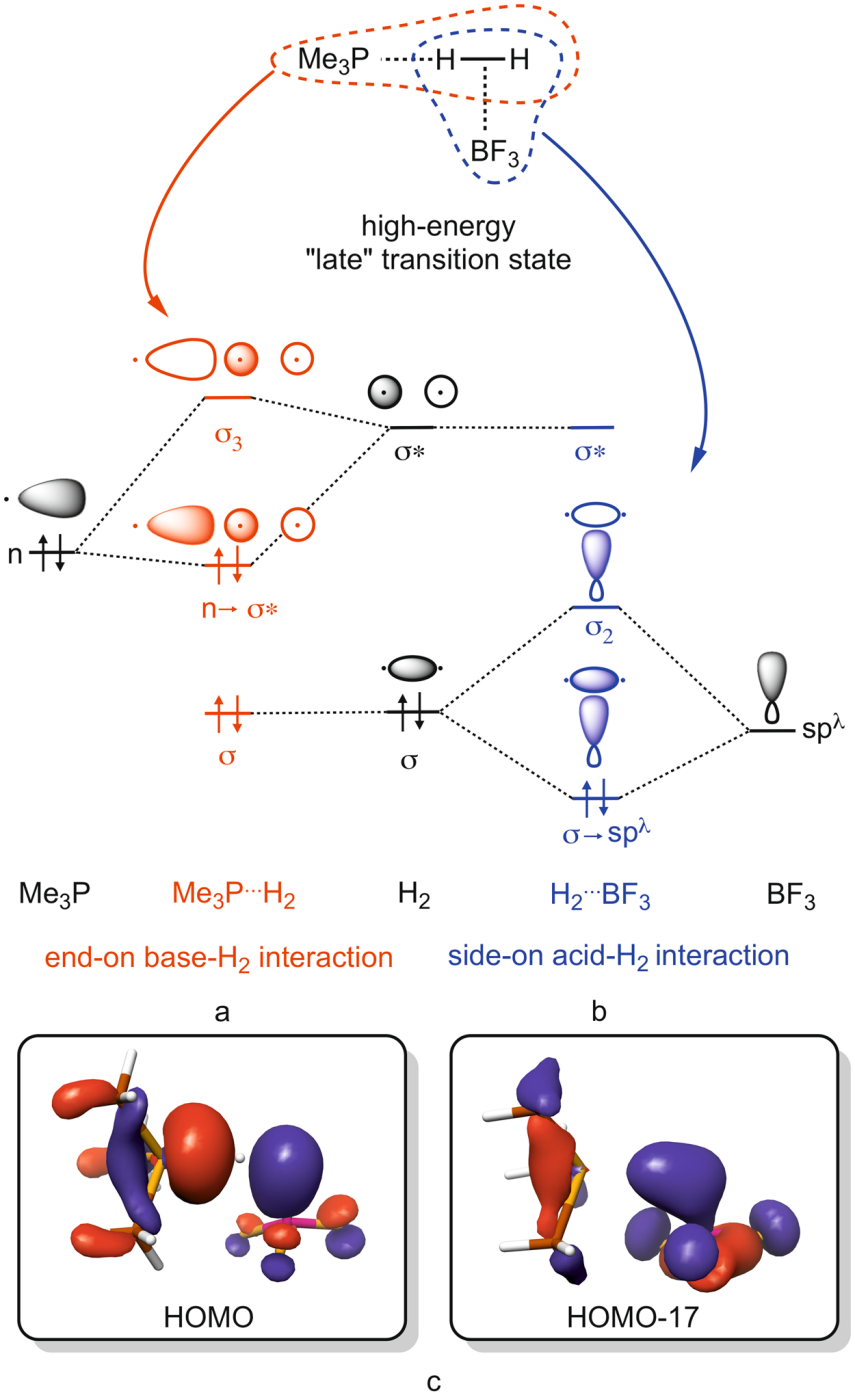
Schematic MO picture of end-on base-H_2_ interaction (**a**) and acid side-on interaction (**b**) in “late” high-energy transition states with arbitrarily chosen fragment orbital energy levels and (**c**) actual MOs of Me_3_P–BF_3_ most resembling to these idealized interactions (see also ESI).

The molecular orbital pictures shown in Fig. 5a and b present the two main charge-control sub-processes that ultimately lead to bond breaking in H_2_. In the end-on base-H_2_ activation mechanism (Fig. 5a) a strong electron donor populates the antibonding σ* orbital of H_2_ (shown in red) formally resulting in a “four-electron – two orbital” filled-filled repulsive interaction along the H–H axis. Note that in this 4e^−^ extreme molecular hydrogen is split formally into two hydrides (H^−…^H^−^). The other single bond breaking mechanistic extreme is removing the electrons from the bonding orbital, represented by the side-on acid-H_2_ interaction in Fig. 5b. In this case, a strong electron acceptor, the Lewis acid, takes most of the electrons of the σ bond of H_2_, with which the H^…^H interaction becomes nonbonding and repulsive due to electrostatic repulsion of the H^+^ centers – in a complete two-electron removal. Formally, two protons are formed in this mechanistic extreme.

As discussed above, a combination of end-on base-H_2_ and the side-on acid-H_2_ extremes, and enhancing cooperative effects form the basis of the current understanding of the reactivity of FLPs towards H_2_. It is important to stress out, however that donation to a high-lying orbital, such as the σ* of H_2_, is a very energy-demanding process in general. This simple concept can be somewhat witnessed, for example, in the lack of interaction of H_2_ with Lewis bases. Accordingly, a significant donation to the high-lying σ*(H_2_) orbital (Fig. 5a) should manifest in a high-energy transition state, which is in striking contrast with the barrierless H_2_ activation processes revealed in Fig. 4 for many systems and, as also implied by many earlier computational studies. In addition, the base^…^H_2_ distance is consistent with a notable base → σ*(H_2_) donation only for Me_3_P–BF_3_ and Mes_3_P–BPh_3_ with high-energy TSs and, also, these are the only structures in which the H_2_ distance is elongated to the extent that is expected for a notably populated σ*(H_2_). These notions imply that only high-energy TSs are consistent, in terms of their structure and energy with an activation that involves donation from the base to the σ*(H_2_). For Me_3_P–BF_3_, also the H–H–B angle of 102.7° is more or less in agreement with the proposed side-on approach of the acid to H_2_. Another interesting dilemma that one faces when trying to imagine H_2_ activation through these idealized sub-processes is how the overall event becomes heterolytic (formally H^+…^H^−^, i.e. asymmetric in terms of electron density distribution) from two homolytic (H^−…^H^−^ and H^+…^H^+^) sub-events that confine symmetric density distribution about H_2_. So far, “cooperativity” has been seen as a satisfying remedy for this controversy, referred to it as the “synergistic nature of the electron donation processes” and quantified as the difference between as the sum of pairwise acid^…^H_2_ and base^…^H_2_ interaction energies and the total three-body base^…^H_2_^…^acid interaction energy^31^.

With a “local” Natural resonance Theory analysis (see ESI) we tried to shed light on how cooperativity in high-energy “late” TSs might be represented in terms of Lewis resonance structures. We found a leading (48%) product-like resonance structure, “Me_3_P^+^–H H–B^−^F_3_”, followed by the reactant-like Lewis structure (34%), “Me_3_P| H–H BF_3_”, augmented by two structures with contribution of about 9% representing donation from the base to H_2_ and donation from H_2_ to the acid. Actually, the mixing of two dominant Lewis structures implies a high degree of delocalization along the P–H–H–B motif in late TSs in good accordance with the delocalized MOs shown in Fig. 5c and in Figure S4, which, we think, might account for the earlier observed cooperative effects.

**Figure 6:**
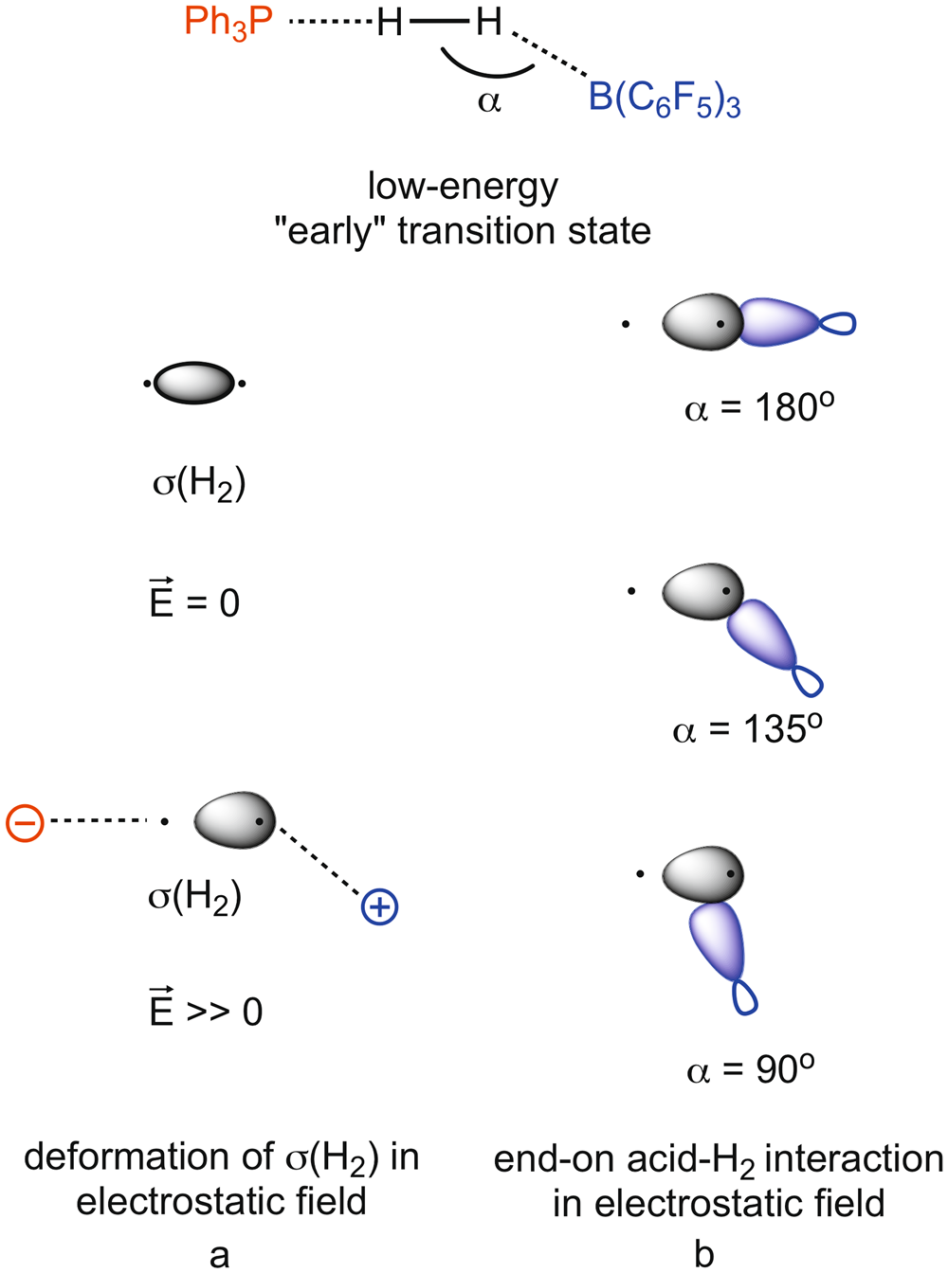
Deformation of the σ(H_2_) orbital in the electrostatic field (E ≫ 0) generated by the acid and the base (**a**) and the MO picture of end-on acid-H_2_ interaction at different angles (α) in this electrostatic field (**b**) in “early” low-energy transition states.

In contrast to high-energy transition states, the geometry revealed for low-energy transition states suggest the lack of base → σ*(H_2_) donation on the one hand, and an end-on approach of the acid to H_2_ on the other hand. Figure 6b shows the schematic MO picture for the latter situation for various H–H–B angles (α = 90°, 135° and 180^o^), termed as end-on acid-H_2_ interaction. The end-on acid-H_2_ interaction, under normal circumstances should be a very inefficient process due to the poor orbital overlap between a non-polarized σ(H_2_) and p-type acceptor orbital (blue in Fig. 6b) of the Lewis acid.

If a strong electrostatic field is, however, generated by the acid and base, it will induce a significant shift of electron density within H_2_ towards the hydrogen that is close to the acid center. As Fig. 6a implies, concomitant with this polarization of H_2_ in the local electrostatic field, even if it is inhomogeneous in the reactive zone, the underlying σ-orbital becomes more centered on the hydrogen that is close to the positive center (acid). This deformation of σ(H_2_) makes the end-on acid-H_2_ interaction (Fig. 6b) competitive in efficiency to that of the side-on approach of the acid (Fig. 5b). Pápai and coworkers recently indeed recognized and articulated the strong polarization of H_2_ in the reaction cavity of FLPs and interpreted these findings with the distortion of σ(H_2_) even in an inhomogeneous field^31^.

The most vital difference from the mechanistic extremes introduced in Fig. 5, i.e. end-on base-H_2_ interaction and side-on acid-H_2_ interaction, is that the density change is only asymmetrical for the electrostatic field assisted end-on acid-H_2_ mechanism presented in Fig. 6, where H_2_ is split into a proton and a hydride (again formally speaking). Such charge separation is energetically very unfavorable in general and, accordingly, the corresponding transition state should be of high energy for “normal” systems (one can easily deduce that for systems with “late” high-energy transition states the “early” charge separated transition state would be even higher in energy). Nonetheless, strong electric fields stabilize this dipolar state of H_2_, which results in a low-energy transition state for systems that can generate the required electrostatic field. Also, this hypothesis clarifies the puzzling small H–H distance in low-energy transition states; first, there is no base → σ*(H_2_) donation that would elongate the hydrogen-hydrogen bond and, second, the asymmetric density shift results in an additional electrostatic attraction between the partially positive and negative hydrogen centers, so their proximity is preferred. Accordingly, the key element of our proposal for the reactivity of H_2_-activating Lewis pairs is that the polarization of H_2_ in the strong electrostatic field generated by certain acids and bases induces a shift in the mechanism from a mixed end-on base/side-on acid-H_2_ activation to an electrostatic field assisted end-on acid-H_2_ activation. These differing electronic effects manifest directly and unmistakably in the structure adopted by the corresponding transition states, resulting in geometrically late or early TSs (vide supra), respectively.

**Figure 7:**
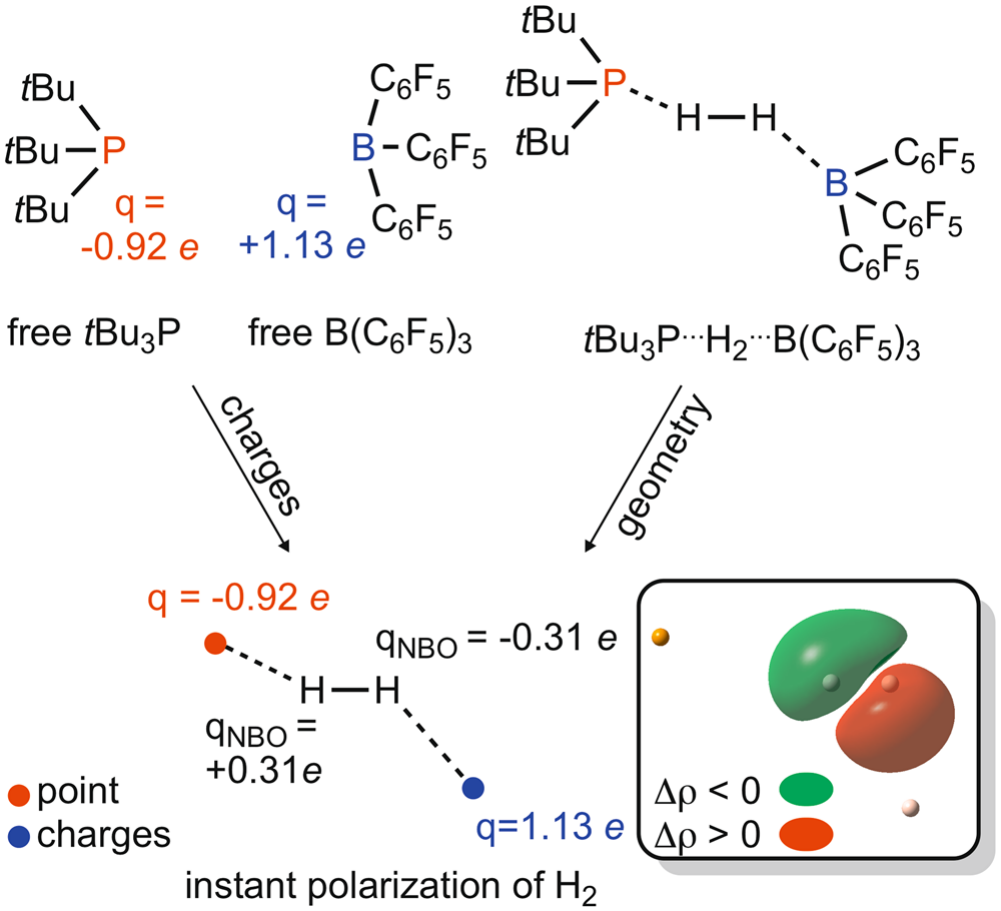
“Instant” charge separation of H_2_ in an idealized electrostatic field generated by the acid and base centers modeled as point charges using their corresponding ChelpG charges in the free reactant state. Δρ in the box shows the deformation of the electron density in the approximate electrostatic field with respect to the electron density of non-polarized H_2_.

Figure 7 illustrates a simple analysis that we carried out to demonstrate that the electrostatic field, even if inhomogeneous, could be strong enough to polarize H_2_ to a great extent even without any charge transfer effect. First we computed the Molecular Electrostatic Potential related ChelpG charges of free *t*Bu_3_P and B(C_6_F_5_), which should give a good estimate for the local electrostatics exerted by the base and acid centers, respectively, in the cavity of the Lewis pair. In Figure S6 we provide support to the view that the cavity of early TSs has exterior-like features respect to H_2_ and, accordingly, MEP derived ChelpG charges offer physically meaningful approximations, even if rather crude ones, for the instant electrostatic effect of the acid and the base. Using the derived charge values (left in Fig. 7) we modeled an approximate field at the geometry of the corresponding transition state (right in Fig. 7) by placing point charges of the same values to the positions of the base and acid centers (bottom in Fig. 7). The distortion of electron density of H_2_ in this field is shown in Fig. 7 with respect to non-polarized H_2_, while its extent was estimated by the atomic charges of the H centers, which was computed to be 0.3 and −0.3 *e* using the standard Natural Population Analysis (NPA) method. This polarization of H_2_ in the cavity of low-energy transition states is significant enough to state confidently that the electrostatic field indeed plays a critical role in the splitting of molecular hydrogen by certain Lewis-pairs.

In order to further support our outlined concept, we carried out a Ziegler-Rauk energy decomposition analysis coupled to a Natural Orbital for Chemical Valence analysis (NOCV) on the investigated transition states. Table 3 lists the results of a Ziegler-Rauk energy decomposition analysis, which separates the interaction energy between three fragments, acid/H–H/base at the transition state geometry, into four physically meaningful terms, such as steric effect (ΔE_Pauli_), charge transfer (ΔE_oi_), electrostatic interaction (ΔV_elst_) and dispersion (E_disp_). The magnitude of these terms clearly highlight that high-energy and low-energy transition states are indeed different in nature. As a matter of fact, our protocol for scrutinizing the origin of activation barriers falls under the umbrella of the so-called Activation Strain Model (ASM), which is a fragment-based approach that aims at separating the energy at any point along the reaction coordinate into the strain energy term, ΔE_strain_, and the interaction energy term, ΔE_int_^83–86^. ΔE_strain_ represents the energy contribution needed to distort the selected fragments, in our case the acid, the base and H_2_, from their equilibrium structure to the geometry they acquire at the transition state. On the other hand, ΔE_int_ accounts for all chemical interaction between the deformed reactants at their positions in the transition state geometry.

**Table 3:** Decomposition of interaction energy between acid^…^H–H^…^base in the transition state of H_2_ cleavage by the investigated systems. All values are in kcal mol^−1^. The relative contributions of stabilizing electrostatic and orbital interactions are given in brackets in percentages.

	ΔE^‡^_app_^PBE^	ΔE_strain_	ΔE_int_	ΔE_Pauli_	ΔV_elst_	ΔE_oi_	E_disp_
Me_3_P–BF_3_	4.8	39.2	−34.4	156.2	−61.2 (33)	−126.2 (67)	−3.3
Me_3_P–B(C_6_F_5_)_3_	−8.2	6.1	−14.3	69.9	−30.7 (42)	−42.2 (58)	−11.2
lut–B(C_6_F_5_)_3_	−6.7	5.0	−11.7	69.3	−30.2 (43)	−39.5 (57)	−11.2
carb · B(C_6_F_5_)_3_	−12.9	4.5	−17.4	62.5	−26.5 (41)	−37.7 (59)	−15.6
*t*Bu_3_P · B(C_6_F_5_)_3_	−10.2	3.6	−13.8	62.7	−27.3 (43)	−35.8 (57)	−13.4
Mes_3_P · BPh_3_	1.8	32.8	−31.0	151.9	−50.2 (27)	−114.8 (73)	−17.9

In general, a much stronger interaction of fragments evolves in high-energy transition states than in LE-TSs. The substantial activation barriers revealed for Me_3_P–BF_3_ and Mes_3_P–BPh_3_ with HE-TSs originate from the deformation of reactants (e.g. ΔE_strain_ = 39.2 kcal mol^−1^ for Mes_3_P–BPh_3_), which seems to be negligible in low-energy transition states. This striking difference in the nature of transition states has been spotted also in earlier studies, however it has never been conceptualized^62^. The relative contributions of stabilizing electrostatic and orbital interactions, given in percentages in Table 3, are more informative in this case. Electrostatic interactions play an enhanced role (~42%) in low-energy transition states in line with the above-proposed mechanistic differences. Also telltale finding is the significant Pauli repulsion in HE-TSs stemming from the overlapping fragment electron densities in the reactive region, indirectly implying charge transfer to the σ*(H_2_) orbital in these cases. Another interesting finding listed in Table 3 is the dispersion between fragments in the transition states. Namely, in the case of systems with bulky substituents, dispersion stabilizes the transition states by about 11–18 kcal mol^−1^ with respect to free reactants. In this context it is worth noting the crucial importance of dispersion in correctly describing and quantitatively understanding contemporary chemical problems, such as C–H bond activations^87^ and Suzuki-Miyaura cross-coupling reactions^82^ amongst many others^88^. Using the Non-Covalent Interaction method^72,73^ we scrutinized the various secondary interactions that lead to this contribution of dispersion and, accordingly, demonstrated the presence of π–π stacking, C–F^…^H and other specific weak-interactions in the transition states and ion-pair products (see Figures S2 and S3).

Interestingly, Bickelhaupt and co-workers arrived to conclusions and concept very similar to what we have presented herein for transition states when investigating the nature of dihydrogen bonding in Lewis base–H/H–acid adducts^89^. Namely, depending on the electronegativity of the base and acid centers, there is a gradual change from covalent to donor-acceptor type interaction between the two fragments, which can be directly monitored in the electrostatic/orbital interaction contributions to the bonding. The most striking similarity to our concept, however, is the demonstrated participation of σ*(H_2_) orbital in the bonding of apolar systems, while this orbital becomes vacant and, accordingly, non-effective in polarized donor-acceptor type bonding, which finding aligns perfectly to our notion for LE-TSs.

**Figure 8:**
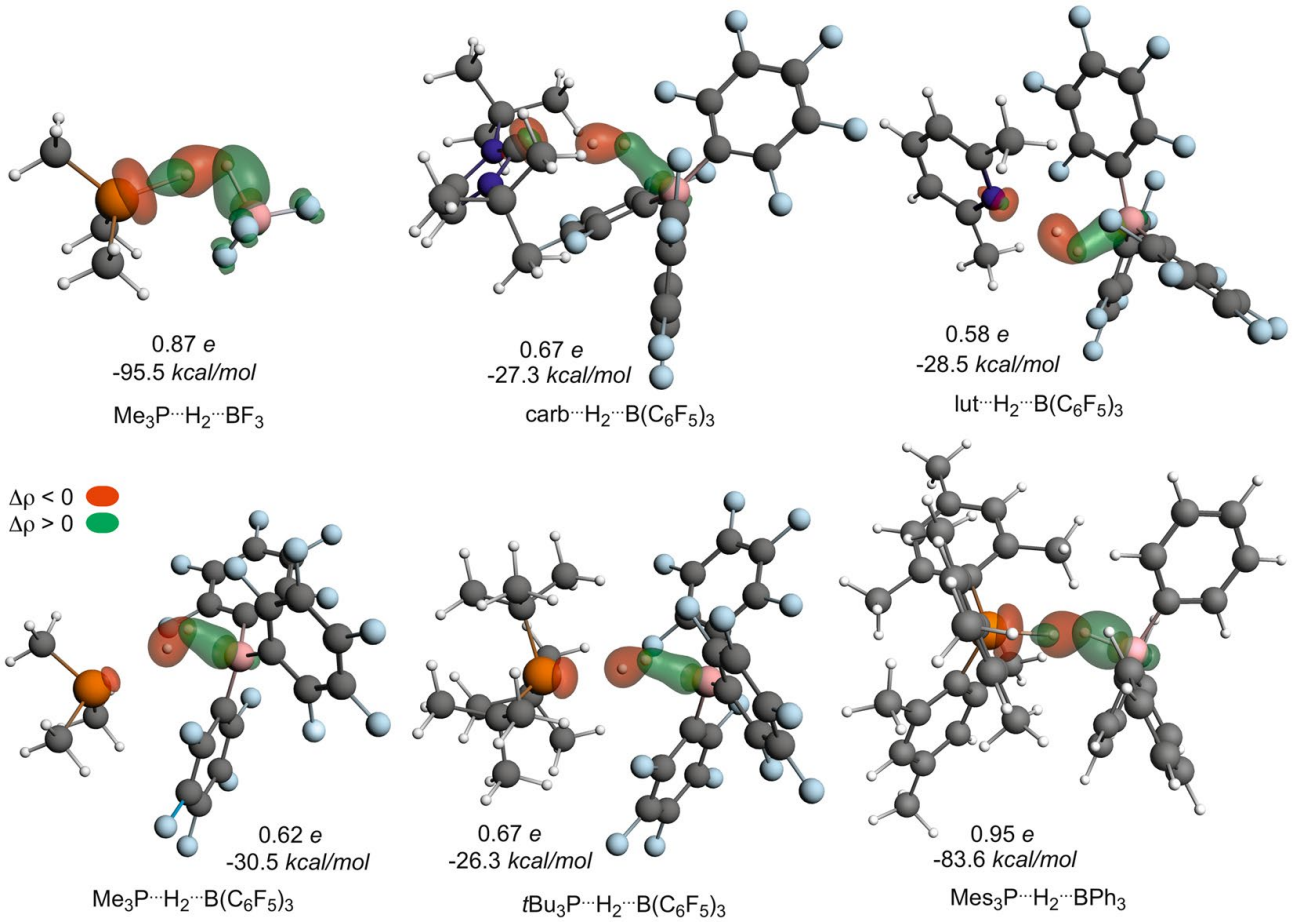
Most dominant NOCVs characterizing the overall density deformation upon transition state formation from acid, H_2_ and base fragments given together with the amount of total density reorganization and corresponding stabilization energy.

The most convincing support for the proposed mechanistic differences is provided by the so-called NOCV orbitals (Fig. 8) constructed from the electron density change upon going from non-interacting to interacting base^…^H–H^…^acid fragments at the transition state geometry. Accordingly, these NOCVs provide a direct visual access to the most important electronic rearrangements as density accumulations (green regions) and depletions (red areas) that take place in the transition states of the investigated systems. The basics and implications of NOCV analysis and its intuitive application have been discussed and showcased for complex chemical problems, such as the trans-effect of ligands^90^, redox non-innocence of ligands^91^ and halogen bonding^92,93^, in which studies this method provided critically new, easily understandable insights. For all systems with a low-energy TS, the density flow is characteristically asymmetric describing an overall charge transfer from σ(H_2_) orbital to the acid center, whereas the base is involved only in a very minor extent into the overall density rearrangement. Most importantly, the most unambiguous evidence of a significant base-to-σ*(H_2_) donation, which is a plane of zero accumulation density (the nodal plane) between the hydrogen centers, cannot be witnessed for these low-energy TSs.

In contrast, the antibonding pattern of σ*(H_2_) can be clearly recognized in the density build-up around H_2_ in the high-energy transition states of Me_3_P–BF_3_ and Mes_3_P–BPh_3_.The latter NOCVs convincingly support the earlier proposed significant electron donation from the lone pair of the base to the σ* orbital of H_2_. Thus, in line with former diagnostics, this NOCV analysis also reveals strict differences between low-energy and high-energy transition states. Namely, then again, the electronic structure rearrangement is consistent with a combination of base → σ*(H_2_) donation and side-on acid-H_2_ activation sub-events for high-energy TSs, whereas it is consistent with an electrostatic field assisted end-on acid-H_2_ activation mechanism for low-energy transition states.

In order to gain further insights into the hypothesized different base-H_2_ interactions and splitting mechanisms in the two types of TSs we analysed the electron density distributions of the studied transition states using NBO analysis, AIM analysis and Bader charge distribution, Wiberg bond indices, local NRT analysis and the Laplacian along the H–H bond. Herein we discuss only the most telltale findings while further, fully conforming details can be found in the ESI. The atomic charges of the base and acid centres and central hydrogen atoms and their change upon going from non-interacting reactants to the TS are especially important in monitoring the charge transfer processes. Both Bader and NBO (given in parentheses) charge analysis is consistent in that in systems with low-energy “early” TSs the charge of the base donor atom barely changes (from −0.031 *e* to 0.052 *e*) when going from the non-interacting reactants to the TSs, whereas the population of the base donor atom drops significantly in high-energy (late) TSs, (by 0.222 *e* in Me_3_P-BF_3_ and 0.272 *e* in Me_3_P-BPh_3_), signaling significant charge transfer from the base to H_2_ in the these cases. Also in agreement with the concept outlined above, donation from H_2_ to the Lewis acid is apparent in all systems resulting in a population increase at the boron center ranging from 0.250 *e* to 0.467 *e*. Polarization, quantified by the charge separation at H_2_ in the TSs is significant in both systems, however, it is somewhat more pronounced in late (0.277 *e* to 322 *e*) than in early (0.179 *e* to 0.275 *e*) TSs.

In line with these findings, the electron density (Table S2) is about three times as high at the base^…^H bond critical point (BCP) in high-energy TSs than in low-energy TSs. Together with the Laplacian (Table S3), which is negative (i.e. charge is concentrated) at the BCP of base^…^H interactions for high-energy TSs and positive (i.e. charge is locally depleted) for low-energy TSs indicating, then again, a forming Lewis base^…^H bond (consistent with base-to-σ*(H_2_) donation) for the HE-TSs whereas the lack of (or very weak) base^…^H_2_ interaction for the LE-TSs. Also, the BCP density value of H^…^H bond is much lower (~0.15 a.u.) for “late” TSs than for “early” low-energy TSs (~0.24 a.u.) and the Laplacian values also significantly more negative (~ −0.95 a.u. *vs*. ~−0.37 a.u.) for the latter. These indices demonstrate different H^…^H bond topology in the two types of TSs: the bond is significantly weaker (lower density and less accumulating) for high-energy TSs than for low-energy TSs. We believe that these findings are in strong favor of the reactivity concept introduced above for low-energy early and high-energy late TSs.

To provide further support to our hypothesis, we computed the activation parameters of another classical Lewis pair, H_3_P–BF_3_. As we observed LE-TSs only with B(C_6_F_5_)_3_ in our studies, we expected that BF_3_ cannot exert an electrostatic field that is strong enough to shift the activation process to the regime of electrostatic field assisted end-on acid-H_2_ mechanism. Accordingly, the activation of H_2_ with H_3_P–BF_3_ is projected to traverse a high-energy transition state with substantially elongated H^…^H bond and short P^…^H and H^…^B distances. Indeed, our calculations revealed an apparent barrier of 36.1 kcal mol^−1^, a H–H distance of 1.37 Å and base^…^H and H^…^acid distances of 1.48 and 1.29 Å, respectively. These energy and structural parameters further support a combined end-on base/side-on acid-H_2_ activation mechanism for this system as well. Moreover, the contribution of reactivity quenching to the activation barrier, i.e. the energy of pair formation (ΔE_PF_), is only −3 kcal mol^−1^, implying that the quenching of reactivity is not exclusively the source of high activation barrier in non-activating systems.

## Conclusions

In conclusion, in this quantum chemical study on the reaction of various classical and frustrated Lewis pairs with molecular hydrogen, we provided convincing support for the central role of the electrostatic field in the heterolytic H_2_ splitting process of FLPs. Most importantly, the profound differences in the activation energy parameters of H_2_-activating and non-activating Lewis pairs question the rationality of the general consensus H_2_-splitting mechanism. Our analyses revealed that the widespread reactivity concept, which explains hydrogen-hydrogen bond breaking by the synchronous electron donation from the base to the σ* of H_2_ and electron withdrawal from the σ orbital of H_2_ to the acid, is valid for high-energy transition states, often termed as “late”, with significantly elongated H–H bond and small base^…^H_2_ distance, which features are the most direct manifestations of a significant base → σ*(H_2_) electron donation. Low-energy transition states, in contrast, feature a characteristically short central H–H bond and rather large base^…^H_2_ and H_2_^…^acid distances, for which these TSs are often termed as “early”. An intuitive Ziegler-Rauk energy decomposition analysis coupled to a Natural Orbital for Chemical Valence examination revealed the lack of base → σ*(H_2_) donation in these systems as well as provided confirmation for a characteristically end-on acid-H_2_ interaction in the corresponding transition states.

Our observations put forward the critical importance of electrostatic field, generated by the acid and base centers in the case of geometrically early low-energy transition states. First, a strong electrostatic field polarizes H_2_ to a great extent and, accordingly, shifts its electron density towards the acid, with which the efficiency of the end-on acid-H_2_ interaction significantly increases. In addition, as the charge separated H_2_ is significantly stabilized in a strong electrostatic field, it does not represent a notable energy penalty and the corresponding transition state can be of low energy. Another advantageous geometrical feature of LE-TSs is the ideal large separation of Lewis acid and base that allows stabilizing dispersion between their bulky substituents, which would lead to steric repulsion in more compact transition states. The detailed analysis of electron densities of transition states provides further strong supports for the notion of characteristically different H_2_-splitting mechanisms in the different transition states.

### Computational Details

All geometry optimizations of Lewis acids, bases and their adducts, transition states, ion pairs and free products were obtained from DFT calculations using the long-range corrected *ω*B97x-D functional^94,95^ coupled to the Dunning-type cc-pVDZ basis^96,97^ as implemented in Gaussian09^98^. Each located stationary point was confirmed to be a local minimum or first order saddle point (for TSs) on the potential energy surface by harmonic vibrational frequency calculations at the same level of theory. Zero-point vibration energies and gas-phase thermodynamic corrections were determined in the ideal gas–rigid rotor–harmonic oscillator approximation at T = 298.15K. Subsequent single point calculations were performed using the triple-ζ cc-pVTZ basis^97^ on the optimized geometries to get refined energies (*E*) for the investigated species. Basis set superposition error (BSSE)^99–101^ correction was approximated and corrected for using the Counterpoise method at the latter *ω*B97x-D/cc-pVTZ level of theory. To take into account solvent effects the continuum SMD^102^ model with benzene as solvent was used at the *ω*B97x-D/cc-pVDZ level of theory.

The Ziegler-Rauk energy decomposition^75,76^ and NOCV^77–80,103^ analyses were performed using the PBE/TZ2P^104–106^ functional/basis set combination as implemented in ADF2013^107^ using the optimized geometries obtained as described above. In these calculations, relativistic effects were taken into account using the Zeroth Order Regular Approximation (ZORA)^104,108,109^, whereas dispersion energy was calculated using the revised DFT-D3 method of Grimme^110^. Recently, we discussed the general applicability of this level of theory, i.e. PBE-D3/TZ2P-ZORA, for post-analyses of various relative energy parameters, e.g. ΔE_PF_, and the respective energy values calculated at this level of theory are denoted as, for example, E^PBE^ ^71^. In good agreement with earlier findings^71,90,92^,^93,111^, the average absolute error of E^PBE^ for ΔE_PF_, ΔE_r,_ ΔE_sep_ and ΔE^‡^ parameters (see ESI) is below 3 kcal mol^−1^ (2.38 kcal mol^−1^) respect to *ω*B97x-D/cc-pVTZ.

### Data Availability

All data generated or analysed during this study are included in this published article (and its Supplementary Information files) and are also available from the corresponding author on reasonable request.

## Electronic supplementary material

Supplementary Information
