# Supplementary material for: Heterolytic Splitting of Molecular Hydrogen by Frustrated and Classical Lewis Pairs: A Unified Reactivity Concept
Source: Sci Rep. 2017 Nov 22;7:16024. doi: 10.1038/s41598-017-16244-1 (PMC5700139; doi:10.1038/s41598-017-16244-1)
Supplement: Supplementary file 1 — Supplementary Information [file 41598_2017_16244_MOESM1_ESM.pdf]

Supporting Information

Heterolytic Splitting of Molecular Hydrogen by Frustrated and Classical Lewis Pairs: a Unified Reactivity Concept

Gabriella Skara[a]*, Freija De Vleeschouwer*[a,*]*, Paul Geerlings*[a]*, Frank De Proft*[a] *and Balazs Pinter*[a,*]

[a] Eenheid Algemene Chemie (ALGC), Member of the QCMM VUB-UGent Alliance Research Group, Vrije Universiteit Brussel (VUB), Pleinlaan 2, B-1050 Brussels, Belgium.

E-mail: pbalazs@vub.ac.be and fdevlees@vub.ac.be

Contents:

I. QTAIM analysis of transition states……………………………………………….….......................2
II. NCI analysis of transition states…….……………………………………………….….......................3
III. NCI analysis of ion-pair products ………………………………………....…………………….....4
IV. MO analysis of transition states……………… ……………………………………………………5
V. NRT analysis of transition states……………… ……………………………………………………6
VI. Assessing the exterior like feature of the "cavity" of FLPs…………………………………………7
VII. Laplacian along the H–H bond in TSs…………………………………………………………..8
Table S1. PBE-D3/TZ2P-ZORA level of theory results……..……………………………………….9
Table S2. Wiberg bond indices in TSs……………….. ……..……………………………………….9
Table S3. Electron density values at the BCPs of TSs……………………………………………….9
Table S4. Laplacian values at the BCPs of TSs……………………………..……………………..9
Table S5. Bader charges in TSs…………………………………………………..…………………...10
Table S6. NBO charges in TSs………………………………………………..………………….....10
VIII. Cartesian coordinates……………………………...…..11

I. QTAIM analysis of TSs at ωB97x-D/cc-pVTZ level of theory.

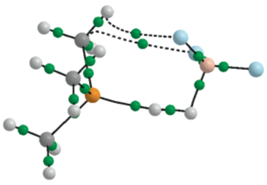

$Me_3P\cdots H_2\cdots BF_3$

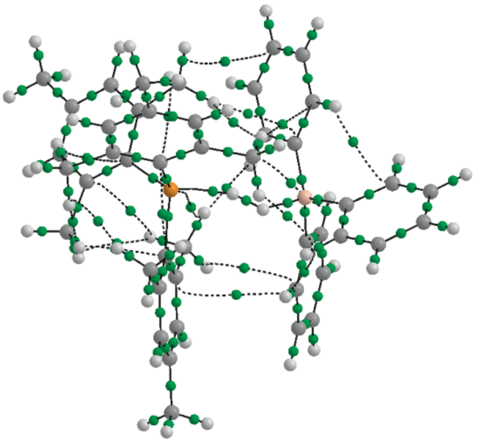

$Mes_3P\cdots H_2\cdots BPh_3$

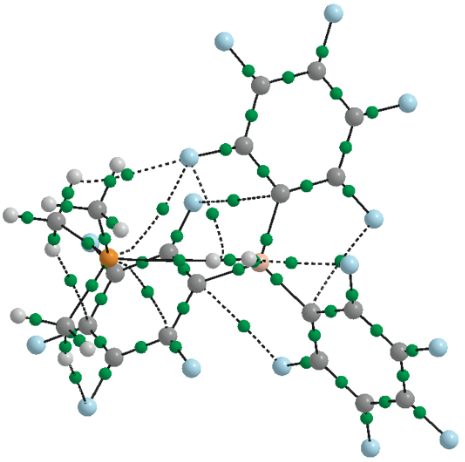

$Me_3P\cdots H_2\cdots B(C_6F_5)_3$

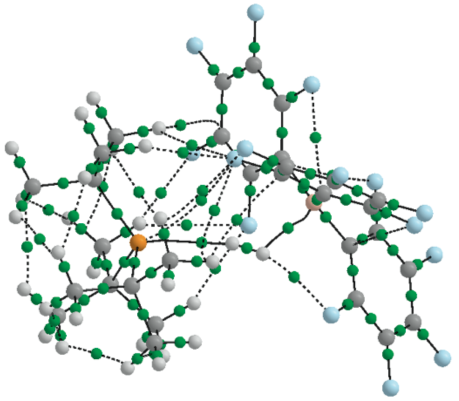

t$Bu_3P\cdots H_2\cdots B(C_6F_5)_3$

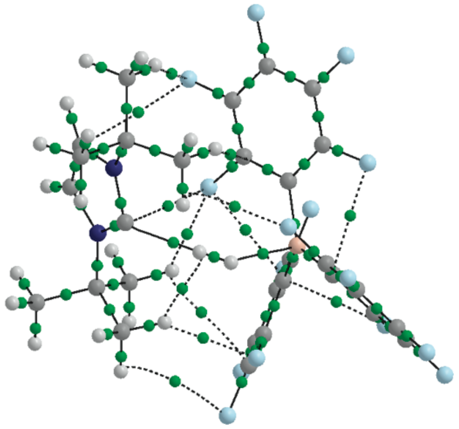

carb$\cdots H_2\cdots B(C_6F_5)_3$

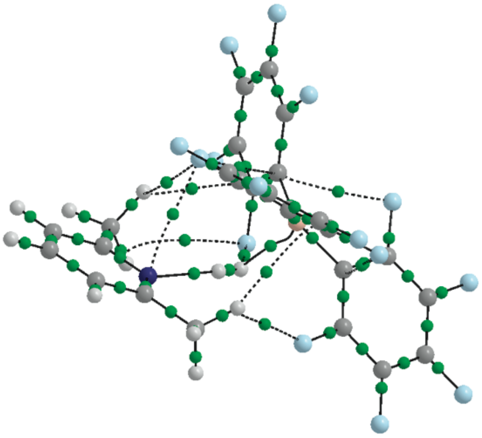

lut$\cdots H_2\cdots B(C_6F_5)_3$

Figure S1. Bond critical points (BCPs, green) and bond paths (lines) in the studied transition states revealed using the AIMAll program. (Version 14.06.21, Todd A. Keith, TK Gristmill Software, Overland Park KS, USA, **2014**, http ://aim.tkgristmill.com)

II. NCI-plots of transition states

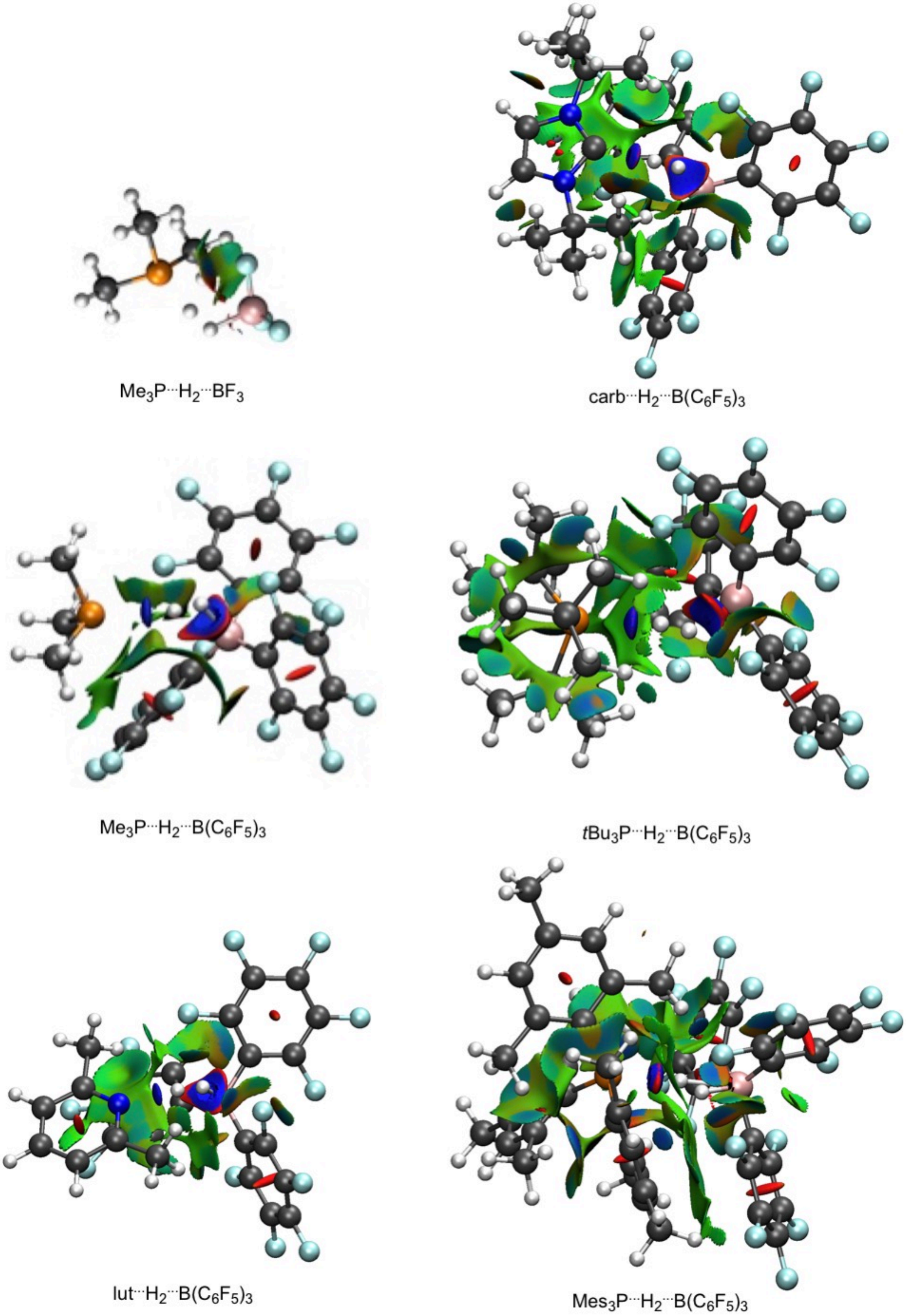

Figure S2. Plots of reduced density gradient isosurfaces with $s = 0.5$ coloured with sign(λ_2)ρ (red – positive (repulsive), blue – negative (attractive) for the investigated transition states.

III. NCI-plots of ion-pair products

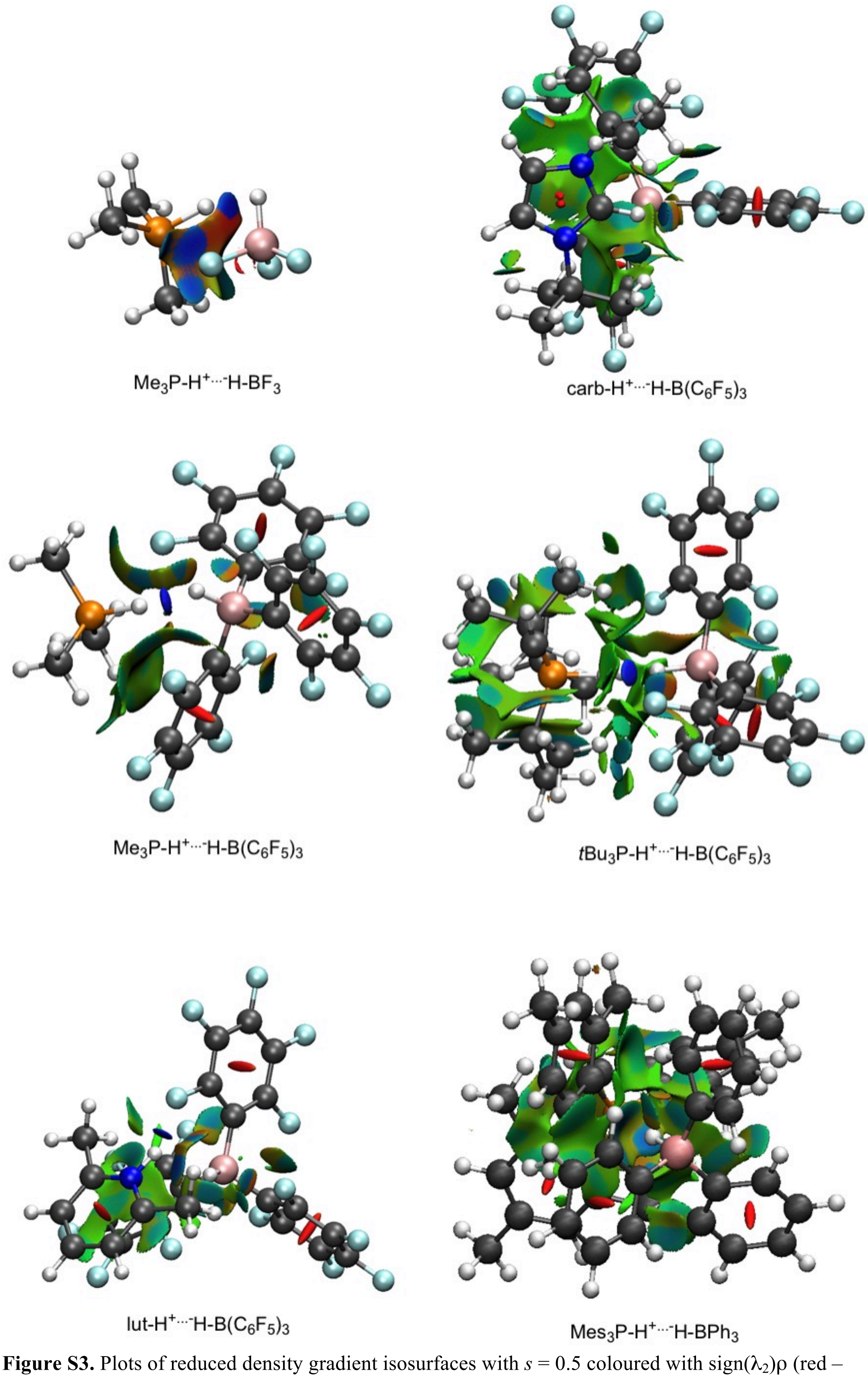

Figure S3. Plots of reduced density gradient isosurfaces with s = 0.5 coloured with sign(λ_2)ρ (red – positive (repulsive), blue – negative (attractive) for the investigated ion-pair products.

IV. MOs of the transition states of $Me_3P–BF_3$ (high energy) and $Me_3P–B(C_6F_5)_3$ (low energy)

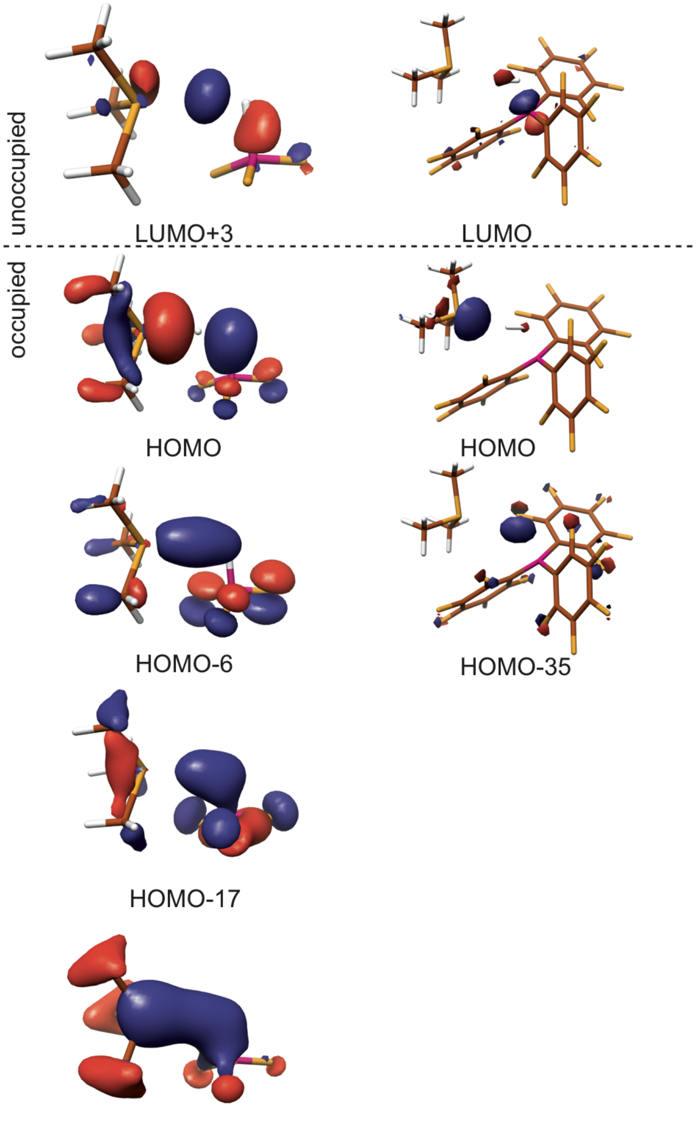

Figure S4. Relevant MOs of the late TS of $Me_3P–BF_3$ (left) and early TS of $Me_3P–B(C_6F_5)_3$ (right).

We believe that the actual MOs for late TSs in Figure S4 can be more intuitively interpreted from the product fragment MOs, than using the reactant fragment orbitals. Accordingly, HOMO of TS-Me_3P-BF_3 might be interpreted as the antibonding bonding combination of an almost formed P–H and B–H bonds, whereas their bonding combination might be best represented by HOMO-6 and HOMO-17 (mixed in with the lone pair representing p orbitals of F's). One of the corresponding antibonding combinations is LUMO+3. "Cooperation" might be seen, according to our interpretation, as the effect of delocalization of the occupied MOs (e.g. HOMO, HOMO-17 and HOMO-19) along the base–H–H–acid core, which of course could not evolve without the presence of *both* the acid and base.

In contrast, in Figure S4, one also can clearly recognize the fragment orbitals in the MOs of early TSs (right), such as the empty p orbital at boron (LUMO), the lone pair of P (HOMO) and s(H_2) (HOMO-25) in the case of $Me_3P–B(C_6F_5)_3$. As such, again, the electronic structure between early and late TSs is strikingly different as shown by these sets of MOs.

V. Natural Resonance Theory analysis of the TSs of $Me_3P–BF_3$ and $Me_3P–B(C_6F_5)_3$

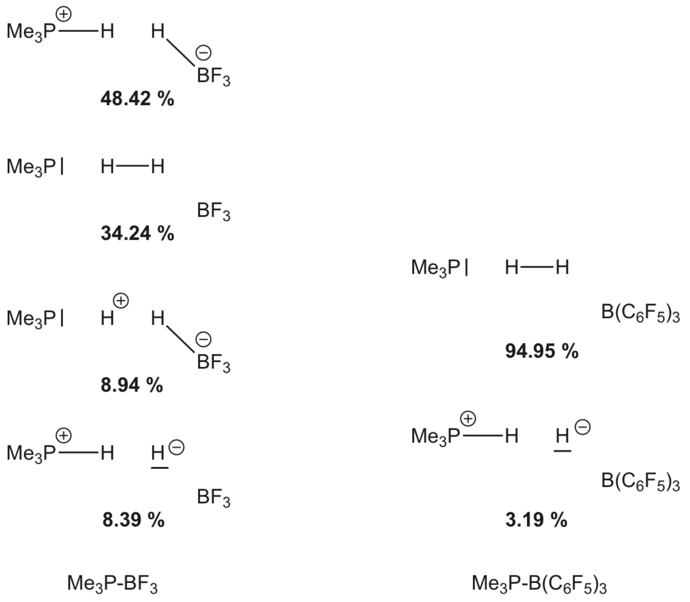

Figure S5. “Local” Natural Resonance Theory (NRT) analysis for the late TS of $Me_3P–BF_3$ (left) and early TS of $Me_3P–B(C_6F_5)_3$ (right).

We carried out a “local” Natural Resonance Theory (NRT) analysis for the HE-TS of $Me_3P–BF_3$ (Figure S5, left) and LE-TS of $Me_3P–B(C_6F_5)_3$ (right) using the implementation of this technique in NBO6 code allowing confined, i.e. „local“ NRT analysis. This NRT analysis is confined to the P–H–H–B moiety, while the many possible Lewis structure “variations” to describe the electron density accurately around the substituents is not considered. On the one hand, the early LE-TS of $Me_3P–B(C_6F_5)_3$ is dominantly represented as a virtual sum of reactant Lewis structures with very minor contribution (3%) from a Lewis extreme that might be rationalized as representing base-to-H_2 donation and/or polarization. In contrast, the reactant-like Lewis structure represents only 34% of the overall structure for late HE-TS of $Me_3P–BF_3$, adding to the leading (48%) product-like Lewis structure. Some contribution (~9%) is also revealed from two structures that might be needed to characterize base to H_2 and H_2 to acid donations and charge transfer-induced polarization at the central H_2 in late TSs.

VI. Assessing the exterior like feature of the “cavity” of FLPs.

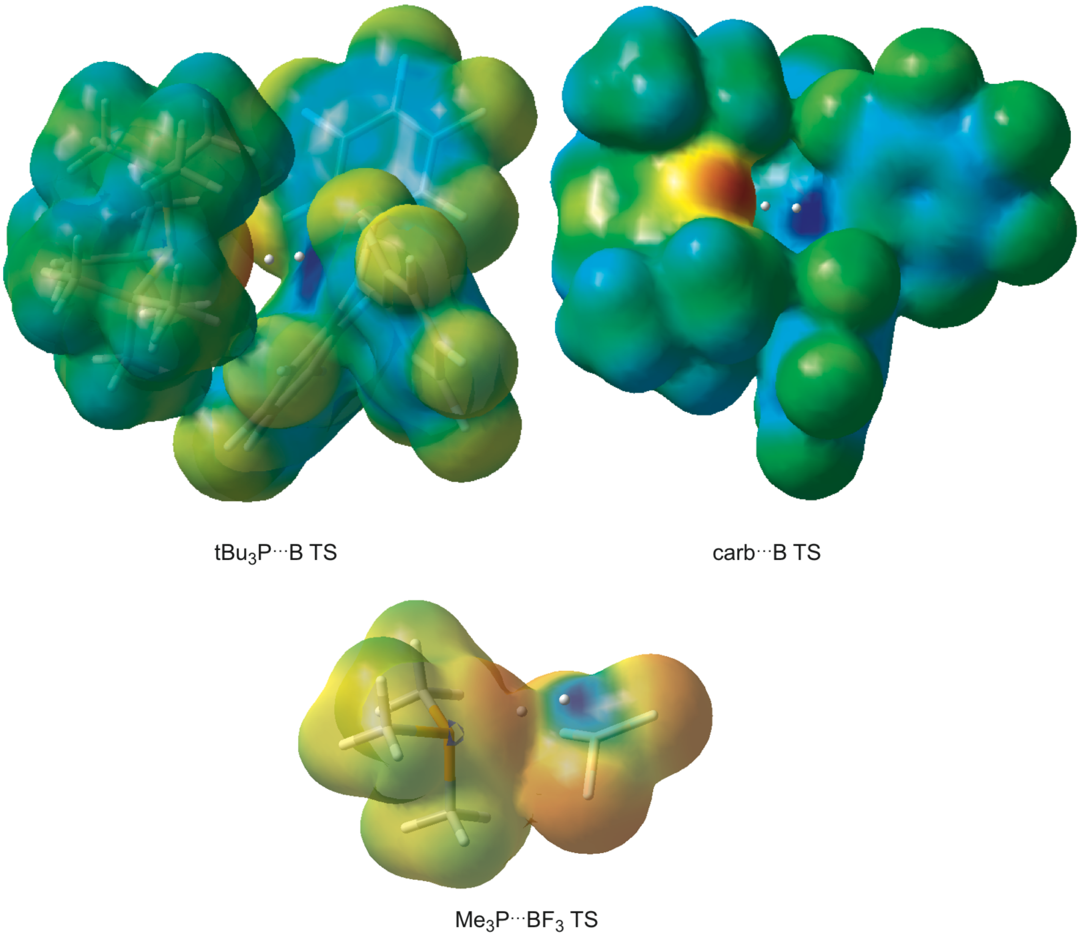

Figure S6. Molecular electrostatic potential surfaces plotted on electron density isosurfaces of 0.005 a.u. showing the “exterior-like” cavity in early TSs (top), implying the validity of approximating the electrostatic effects with MEP derived ChelpG charges. The same surface is plotted for a high-energy TS (bottom), where this approximation is not valid.

With Figure S6 we aim to provide support that the cavity in early TSs have exterior-like properties and using ChelpG charges is a rough, but not incorrect, way of approximating the electrostatic effect of Lewis base and acid centers. For these plots we used the relaxed wavefunction of acid···base at the transition state geometry without the presence of H_2, ensuring practically no interaction between acid and base. The position of H_2 is then showed as in the TSs. The density isosurface selected for these plots is one of its used typical values of 0.005 a.u. mapped with the electrostatic potential. According to the revealed plots (Figure S6, top) we believe that it is realistic to use ChelpG charges for early TSs to approximate the effect of the *intrinsic* force field on H_2 polarization, which excludes the effect of any charge transfer.

VII. Laplacian of the electron density in the B–H–H plane for low- and high-energy TSs.

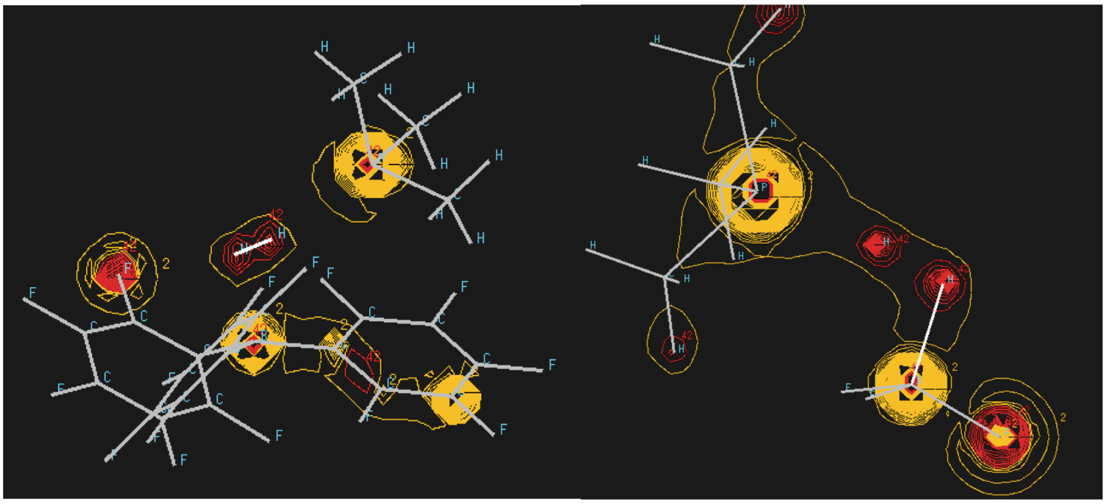

tBu$_3$P···B TS Me$_3$P···BF$_3$ TS

Figure S7. Laplacian of the electron density ($-\nabla^2\rho$) in the B–H–H plane for a low-energy ($Me_3P–B(C_6F_5)_3$, left) and for a high-energy ($Me_3P–BF_3$, right) TS.

Figure S7 shows the Laplacian in the B–H–H plane for a low-energy and a high-energy transition state using the same technical setup with contour line steps of 0.5 a.u.. Charge accumulation (red, $-\nabla^2\rho < 0$) is apparent around the H centers in both structures, however, the charge accumulation is much more pronounced at the hydrogen centers in high-energy TSs, and much less in the internuclear region. Beyond polarization, this finding somewhat implies the formal involvement of $\sigma^*(H_2)$, as the antibonding combinations of MOs are typically more localized on the nuclei and much less in the internuclear region.

Table S1. Activation energy, pair formation energy, reaction energy and separation energy computed at the PBE-D3/TZ2P-ZORA level of theory in kcal mol^{-1}.

	ΔE_{PF}	$\Delta E^{\ddagger}$	ΔE_r	ΔE_{sep}
$Me_3P–BF_3$	-17.6	22.5	19.7	93.8
Me_3P–B	-30.3	22.2	10.6	74.7
lut–B	-12.8	6.1	-13.8	76.0
carb•B	-9.2	-3.7	-48.7	68.4
tBu_3P•B	-10.6	0.4	-21.2	69.0
Mes_3P•BPh_3	-12.5	14.4	11.7	69.4

Table S2. Wiberg indices computed for acid···H base···H and H···H interactions in the transition states at the wB97x-D/cc-pVTZ level of theory. Note the characteristic differences between high-energy ($Me_3P–BF_3$ and Mes_3P•BPh_3) and low-energy TSs.

	base···H	H···H	acid···H
$Me_3P–BF_3$	0.792	0.121	0.729
$Me_3P–B(C_6F_5)_3$	0.106	0.690	0.235
lut–$B(C_6F_5)_3$	0.071	0.724	0.210
carb•$B(C_6F_5)_3$	0.138	0.752	0.147
tBu_3P•$B(C_6F_5)_3$	0.113	0.747	0.173
Mes_3P•BPh_3	0.454	0.373	0.493

Table S3. Electron density values st the BCPs of acid···H, H···H and H···base interactions in the transition states (see Figure S1). Note the characteristic differences between high-energy ($Me_3P–BF_3$ and Mes_3P•BPh_3) and low-energy TSs.

ρ(BCP)	base···H	H···H	acid···H
$Me_3P–BF_3$	0.10	0.15	0.10
$Me_3P–B(C_6F_5)_3$	0.03	0.24	0.05
lut–$B(C_6F_5)_3$	0.03	0.24	0.04
carb•$B(C_6F_5)_3$	0.04	0.23	0.03
tBu_3P•$B(C_6F_5)_3$	0.03	0.24	0.04
Mes_3P•BPh_3	0.09	0.15	0.09

Table S4. Laplacian values at the BCP of acid···H, H···H and H···base interactions in the transition states. Note the characteristic differences between high-energy ($Me_3P–BF_3$ and Mes_3P•BPh_3) and low-energy TSs.

$-\nabla^2\rho$(BCP)	base···H	H···H	acid···H
$Me_3P–BF_3$	-0.14	-0.35	-0.08
$Me_3P–B(C_6F_5)_3$	0.04	-0.96	0.03
lut–$B(C_6F_5)_3$	0.07	-0.98	0.04
carb•$B(C_6F_5)_3$	0.05	-0.91	0.04
tBu_3P•$B(C_6F_5)_3$	0.04	-0.96	0.04
Mes_3P•BPh_3	-0.11	-0.39	0.06

Table S5. Bader charge at the base center, H1 (closer to the base), H2 and the acid center given in *e* . In brackets are given the change of these charges with respect to free reactants to monitor charge tranfsers.

	base center	H1	H2	acid center
$Me_3P–BF_3$	1.65	0.25	-0.45	2.36
	(0.15)	(0.25)	(-0.45)	(-0.12)
$Me_3P–B(C_6F_5)_3$	1.46	0.16	-0.13	1.93
	(-0.04)	(0.16)	(-0.13)	(-0.09)
lut–$B(C_6F_5)_3$	-1.10	0.20	-0.16	1.94
	(0.08)	(0.20)	(-0.16)	(-0.08)
carb•$B(C_6F_5)_3$	0.69	0.19	-0.19	1.95
	(-0.06)	(0.19)	(-0.19)	(-0.07)
tBu_3P•$B(C_6F_5)_3$	0.86	0.15	-0.15	1.94
	(-0.01)	(0.15)	(-0.15)	(-0.08)
Mes_3P•BPh_3	1.59	0.21	-0.39	1.85
	(0.09)	(0.21)	(-0.39)	(-0.14)

Table S6. NBO charge at the base center, H1 (closer to the base), H2 and the acid center given in *e* In brackets are given the change of these charges with respect to free reactants to monitor charge tranfsers.

	base center	H1	H2	acid center
$Me_3P–BF_3$	1.05	0.12	-0.16	1.16
	(0.28)	(0.12)	(-0.16)	(-0.25)
$Me_3P–B(C_6F_5)_3$	0.82	0.17	-0.01	0.66
	(0.05)	(0.17)	(-0.01)	(-0.47)
lut–$B(C_6F_5)_3$	-0.50	0.20	-0.05	0.72
	(-0.03)	(0.20)	(-0.05)	(-0.41)
carb•$B(C_6F_5)_3$	0.12	0.16	-0.12	0.81
	(0.05)	(0.16)	(-0.12)	(-0.32)
tBu_3P•$B(C_6F_5)_3$	0.84	0.15	-0.07	0.76
	(0.05)	(0.15)	(-0.07)	(-0.37)
Mes_3P•BPh_3	1.05	0.20	-0.12	0.45
	(0.22)	(0.20)	(-0.12)	(-0.42)

VIII. Cartesian coordinates of optimized structures

$B(C_6F_5)_3$

B	0.00037300	-0.00013900	0.00082100
C	-0.64120500	1.43056000	0.00121100
C	-0.10107500	2.48235500	-0.74498600
C	-1.78633200	1.72746200	0.74649400
C	-0.66191900	3.75133100	-0.76580900
F	0.98028200	2.28163500	-1.50267000
C	-2.36117400	2.99014500	0.76588100
F	-2.35637900	0.78695200	1.50419300
C	-1.79536600	4.00406200	-0.00011000
F	-0.13089600	4.72104700	-1.50314800
F	-3.43943600	3.23861800	1.50185400
F	-2.33725500	5.21204400	-0.00106400
C	1.56005300	-0.15997000	0.00082100
C	2.20077700	-1.15506300	-0.74346200
C	2.38970500	0.68437400	0.74471700
C	3.58025800	-1.30376400	-0.76432000
F	1.48628700	-1.99324900	-1.49885100
C	3.77075000	0.55103900	0.76416000
F	1.85972500	1.64908600	1.50110700
C	4.36601100	-0.44673600	-0.00071400
F	4.15442700	-2.25041400	-1.49937900
F	4.52514700	1.36144400	1.49913400
F	5.68308000	-0.58136200	-0.00128600
C	-0.91812500	-1.27083500	0.00062100
C	-0.60284100	-2.41132100	0.74526500
C	-2.09949100	-1.32820600	-0.74483400
C	-1.40931900	-3.54033300	0.76464300
F	0.49656900	-2.43475600	1.50312200
C	-2.91855400	-2.44808800	-0.76574300
F	-2.46615700	-0.29084000	-1.50186000
C	-2.57054700	-3.55666000	-0.00098300
F	-1.08579000	-4.59871200	1.50035100
F	-4.02458100	-2.47212200	-1.50209700
F	-3.34629800	-4.62952800	-0.00176600

BF_3

B	0.00000000	0.00000000	0.00000000
F	0.00000000	1.32172700	0.00000000
F	1.14464900	-0.66086400	0.00000000
F	-1.14464900	-0.66086400	0.00000000

BPh_3

C	-1.94906800	1.58369500	0.66164000
C	-0.72052000	1.39432500	-0.00021900
C	-0.16473600	2.50639600	-0.66179200
C	-0.80870600	3.74127900	-0.67636500
C	-2.01742100	3.90309600	0.00031500
C	-2.58418800	2.82313700	0.67672500
B	0.00020900	-0.00002700	-0.00033200
C	1.56812100	-0.07314600	-0.00024100
C	2.25317900	-1.11069000	-0.66165000
C	3.64459400	-1.17069300	-0.67618500
C	4.38926500	-0.20483800	0.00029600
C	3.73754200	0.82622800	0.67646200
C	2.34658000	0.89613900	0.66137600
C	-0.84718100	-1.32124100	-0.00029700
C	-2.08819600	-1.39547900	-0.66183400
C	-2.83608500	-2.57034000	-0.67643500
C	-2.37228600	-3.69823300	0.00016900
C	-1.15358100	-3.64956500	0.67654300
C	-0.39733700	-2.48007900	0.66149400
H	0.56102300	-2.45778200	1.18602300
H	-0.78933900	-4.52894600	1.21141000
H	-2.96202400	-4.61735000	0.00039000
H	-3.78742600	-2.60582900	-1.21100900
H	-2.46744100	-0.51505400	-1.18633100
H	1.68067900	-1.87946700	-1.18631800
H	4.15117600	-1.97677700	-1.21065600
H	5.48013300	-0.25578300	0.00053600
H	4.31679000	1.58162800	1.21116000
H	1.84787700	1.71491700	1.18574000
H	0.78728300	2.39496800	-1.18649800
H	-0.36415500	4.58306200	-1.21100200
H	-2.51893300	4.87318300	0.00050800
H	-3.52793100	2.94703200	1.21156900
H	-2.40862100	0.74239000	1.18612600

carb

C	-0.00000100	-0.55713400	-0.00002200
C	0.67748900	1.62425600	-0.01354600
H	1.36558200	2.46104600	-0.02654400
C	-0.67750000	1.62425300	0.01351800
H	-1.36560800	2.46102800	0.02654200
N	1.06556300	0.29189800	-0.02661200
N	-1.06555800	0.29189300	0.02657000
C	2.45685400	-0.22152400	0.00150800
C	-2.45685300	-0.22151900	-0.00149800
C	-3.44973000	0.92532400	0.19456700
H	-4.46906400	0.51390300	0.21740400
H	-3.27777500	1.45095400	1.14628700
H	-3.40613500	1.65538600	-0.62785700
C	-2.68793900	-0.88884500	-1.36216000
H	-3.70416300	-1.30855100	-1.41983900
H	-2.56556500	-0.15691500	-2.17565400
H	-1.95639100	-1.69540600	-1.50960000
C	-2.61718600	-1.24166400	1.13028900
H	-1.87074400	-2.03961900	1.02498400
H	-2.46893800	-0.75845800	2.10836800
H	-3.62675300	-1.67958700	1.10467100
C	2.68794400	-0.88874700	1.36222000
H	2.56560900	-0.15675400	2.17565800
H	1.95638100	-1.69527900	1.50971500
H	3.70415800	-1.30848200	1.41989500
C	2.61720000	-1.24173300	-1.13021900
H	2.46889500	-0.75860100	-2.10832600
H	3.62679100	-1.67960800	-1.10462900
H	1.87081800	-2.03973500	-1.02485800
C	3.44971200	0.92531900	-0.19463200
H	4.46905600	0.51391500	-0.21737100
H	3.27779200	1.45083900	-1.14641900
H	3.40607400	1.65547200	0.62771300

lut

N	0.00000000	0.94494700	0.00000200
C	1.15339500	0.26536700	-0.00610800
C	1.19792200	-1.13240800	-0.00869600
C	0.00000000	-1.83861500	-0.00000100
C	-1.19792200	-1.13240800	0.00869600
C	-1.15339500	0.26536800	0.00611100
C	2.40992300	1.09431500	0.00355300
C	-2.40992300	1.09431600	-0.00355500
H	-0.00000100	-2.93062600	-0.00000200
H	2.15685300	-1.65285500	-0.01576000
H	-2.15685300	-1.65285400	0.01576000
H	-2.52021300	1.60067700	-0.97511800
H	-2.35004400	1.87799100	0.76471700
H	-3.30646300	0.48437900	0.17329000
H	2.35006700	1.87795100	-0.76476200
H	2.52018400	1.60072700	0.97509400
H	3.30646900	0.48437000	-0.17323600

Me_3P

P	-0.00001000	-0.00007900	-0.60597900
C	-1.43195600	-0.78357100	0.28033500
H	-1.32656500	-0.72424100	1.37638800
H	-2.36622800	-0.28223800	-0.01530500
H	-1.51152800	-1.84158500	-0.01296700
C	0.03728700	1.63177800	0.28034700

H	-0.84001600	2.22890700	-0.01213500
H	0.03752700	1.51135400	1.37647600
H	0.93780500	2.19072700	-0.01693600
C	1.39460600	-0.84814000	0.28039000
H	1.29084300	-0.78690600	1.37650900
H	2.35027000	-0.38739900	-0.01312500
H	1.42842300	-1.90782800	-0.01566000

==

tBu_3P

==

P	0.00048000	-0.00075800	-0.71963900
C	-1.26359000	-1.27526300	-0.00435000
C	1.73747700	-0.45704100	-0.00453400
C	-0.47318700	1.73110800	-0.00470200
C	0.70067800	2.70326600	-0.21901900
H	0.35015400	3.72995000	-0.01999500
H	1.54294800	2.51348200	0.45849900
H	1.07042800	2.67613600	-1.25543600
C	-0.89355900	1.80059400	1.47030900
H	-1.08226800	2.85291100	1.74670000
H	-1.82114900	1.24706500	1.66823100
H	-0.11842600	1.41601500	2.14653100
C	-1.61741500	2.27675800	-0.88527100
H	-1.81208300	3.32870100	-0.61433200
H	-1.34536300	2.24699300	-1.95138900
H	-2.55960300	1.73157300	-0.76235300
C	2.00446300	-0.13106700	1.47144200
H	3.01236500	-0.48659700	1.74919700
H	1.97969700	0.94852300	1.67160500
H	1.28590000	-0.61663900	2.14500500
C	1.99309000	-1.95860800	-0.22441000
H	1.79345900	-2.25791800	-1.26478200
H	3.05557100	-2.17122100	-0.01807200
H	1.40076900	-2.59710400	0.44332200
C	2.78247000	0.26572300	-0.88075400
H	2.62087100	0.05103100	-1.94798700
H	2.77895700	1.35345700	-0.75102900
H	3.79110900	-0.09168800	-0.61091300
C	-1.11032500	-1.67222400	1.47049200
H	-1.92716000	-2.35968100	1.75237100
H	-0.16719900	-2.20015000	1.66507500
H	-1.15953300	-0.80703100	2.14500200
C	-1.16820400	-2.54011200	-0.88348500
H	-1.96777300	-3.24279100	-0.59247900
H	-1.30436400	-2.29435700	-1.94751200
H	-0.21624600	-3.07182100	-0.77937500
C	-2.69183300	-0.74310700	-0.21735600
H	-2.94815300	0.08171300	0.45959300
H	-2.85354200	-0.41067300	-1.25406700
H	-3.40696200	-1.55866000	-0.01699400

==

Mes_3P

==

C	-0.16828900	2.73216300	0.96142100
C	0.44053900	1.67612900	0.23644800
C	1.36772900	1.99236700	-0.78183500
C	1.67682000	3.33526800	-1.02472500
C	1.09198900	4.38162300	-0.31731100
C	0.15811500	4.05524500	0.66453300
P	-0.00511000	-0.00705200	0.85933100
C	1.23736600	-1.22942900	0.24252400
C	2.46003500	-1.21034000	0.96037700
C	3.45363400	-2.14299800	0.66192400
C	3.27701200	-3.12208800	-0.31380500
C	2.07255800	-3.12685300	-1.01228800
C	1.05307400	-2.20104000	-0.76699400
C	2.75014700	-0.17674700	2.02105700
C	4.34449400	-4.14858300	-0.58669100
C	-0.16893300	-2.27917500	-1.64876600
C	2.02396200	0.96982600	-1.67625000
C	1.46121900	5.81588100	-0.58930700
C	-1.20631200	2.47721600	2.02693300
C	-1.68502400	-0.46106900	0.23313200
C	-2.41955700	0.18508900	-0.78551800
C	-3.73932100	-0.21389000	-1.02767200
C	-4.35701500	-1.23992500	-0.31939800
C	-3.61019600	-1.88638300	0.66476600
C	-2.30023400	-1.51214700	0.96061200
C	-5.78295300	-1.63864600	-0.59377800
C	-1.85796300	1.25997800	-1.68294300
C	-1.56286300	-2.27816400	2.03174300
H	2.39335000	3.56850900	-1.81707100
H	-0.33331200	4.85652800	1.22312500
H	4.39592800	-2.10274600	1.21503200
H	1.92032100	-3.87072700	-1.79925400
H	-4.29706000	0.29117100	-1.82108200
H	-4.06207300	-2.70937400	1.22540100
H	2.79040200	0.83875100	1.59527900
H	1.96500300	-0.16203800	2.79188800
H	3.71542700	-0.37706700	2.50534300
H	-1.04913800	-2.66436500	-1.11400000
H	-0.45159300	-1.29349800	-2.04320000
H	0.02448200	-2.94367000	-2.50205600
H	5.35104300	-3.72051700	-0.47300800
H	4.26464300	-4.99124000	0.11929500
H	4.25797700	-4.55992000	-1.60247200
H	-0.69378600	-2.81522800	1.61911300
H	-1.17316800	-1.60349000	2.80885500
H	-2.22268000	-3.01770000	2.50520300
H	-1.72970000	2.21932300	-1.16124600
H	-0.87085100	0.98560800	-2.07894100
H	-2.53043600	1.42609300	-2.53562900
H	-5.88629300	-2.73208900	-0.65998400
H	-6.44880600	-1.29648000	0.21468300
H	-6.14993300	-1.20330400	-1.53370600
H	-2.10961500	2.00719800	1.60607900
H	-0.82793500	1.79099900	2.79936700
H	-1.50798200	3.41738000	2.50796200
H	1.30223000	0.23366600	-2.05451700
H	2.48562400	1.46709300	-2.54030200
H	2.80758200	0.39886100	-1.15680500
H	0.58009200	6.47279400	-0.54454900
H	2.18074800	6.18274200	0.16065700
H	1.92593400	5.93266800	-1.57855700

==

H_2

==

H	0.00000000	0.00000000	0.37930000
H	0.00000000	0.00000000	-0.37930000

==

carb–$B(C_6F_5)_3$

==

C	-2.04467900	-0.84452300	-3.24028300
C	-0.88107300	-0.31028200	-2.70623600
C	-0.27467800	-0.81228700	-1.54854700
C	-0.91585200	-1.90321700	-0.94862700
C	-2.07113400	-2.46970700	-1.46536700
C	-2.64481800	-1.92617200	-2.60764400
B	1.04434100	-0.19490800	-0.97063700
C	2.08871700	-1.11368300	-0.23754900
C	2.35361400	-2.42485300	-0.64531000
C	3.22789700	-3.26246500	0.03188100
C	3.86084000	-2.79822200	1.18039900
C	3.63372300	-1.49957700	1.61954700
C	2.78163700	-0.67940700	0.89533500
F	1.76754600	-2.91800100	-1.73929900
F	2.61257700	0.56251800	1.35870000
F	4.22229300	-1.05602000	2.72728300
F	4.68113300	-3.59223900	1.85171000
F	3.45843000	-4.49964700	-0.39648100
F	-0.33286000	0.71296700	-3.36663700
F	-0.43542300	-2.44620500	0.16855500
F	-2.64481900	-3.51085400	-0.86845300
F	-3.77465000	-2.42935500	-3.08382800
F	-2.59583000	-0.32712100	-4.33481000
C	1.34240800	1.34121200	-1.11056000
C	2.62977500	1.81980100	-1.37191000
C	2.92449900	3.17200300	-1.46852600
C	1.90818600	4.10246000	-1.28049500
C	0.61272000	3.67308500	-1.01531400

C	0.34912400	2.31215900	-0.95357600
F	3.63820000	0.96416800	-1.56350800
F	-0.90775200	1.95408800	-0.69211400
F	-0.35607500	4.56386400	-0.82646700
F	2.17477800	5.39799200	-1.35555600
F	4.16084700	3.58487600	-1.73257700
C	-4.53045600	-1.47494500	0.76533700
C	-4.48813300	0.05605500	0.77857200
C	-3.87947800	0.59552800	-0.51860800
N	-3.62139700	0.47824900	1.90548700
C	-4.03907800	1.11769100	3.06276600
C	-2.93504300	1.26863400	3.83212600
N	-1.88543100	0.70148300	3.12262800
C	-2.28391200	0.20990100	1.91526100
C	-0.45931700	0.75503000	3.52184000
C	-0.34135200	0.95839300	5.03453400
C	-5.89998700	0.61669000	0.95253900
C	0.18784400	1.93070900	2.78140300
C	0.20968800	-0.56738400	3.13922800
H	-2.83366200	1.72178100	4.81087100
H	-5.06276700	1.41169300	3.26046800
H	-5.03489900	-1.85657100	1.66641900
H	-5.06987700	-1.84500100	-0.12027500
H	-3.50923900	-1.87543900	0.75657400
H	-5.89712900	1.71610500	1.00422000
H	-6.50811800	0.32191200	0.08510400
H	-6.39318100	0.21840400	1.85200400
H	-4.46265200	0.25048500	-1.38688200
H	-3.87144600	1.69588200	-0.51503100
H	-0.31264200	2.87464100	3.04770700
H	1.25535600	2.01411100	3.03077000
H	0.08449500	1.78324000	1.69850500
H	1.27980000	-0.52474300	3.39183300
H	-0.24976200	-1.40433900	3.68636000
H	0.08850800	-0.76769100	2.06782100
H	-0.89109600	0.18104700	5.58659300
H	0.71825500	0.89501000	5.32102200
H	-0.70768300	1.94574800	5.35284900
H	-2.84181000	0.25509000	-0.61248700

==

lut–$B(C_6F_5)_3$

==

N	0.44667900	-0.44695500	2.03085200
B	0.18074100	-0.10172300	0.40844800
F	-1.74298000	-2.12236500	1.78106700
F	-3.68522100	-3.43035600	0.56022900
F	-4.07460800	-3.17119500	-2.12751400
F	-2.44128100	-1.52405800	-3.56436800
F	-0.48678600	-0.18070900	-2.35880700
F	2.42116200	1.52325200	1.18665900
F	4.90800000	1.46506100	0.26675500
F	5.61525900	-0.29511400	-1.69872400
F	3.74896100	-2.02313400	-2.67739900
F	1.29168300	-2.03735400	-1.74266000
F	-2.35795300	0.70419300	1.51805500
F	-3.81925900	2.83799700	0.87622500
F	-2.84760600	4.69609200	-0.87325900
F	-0.37947300	4.34142800	-1.96758700
F	1.08233600	2.23625000	-1.36987600
C	-0.96609800	-1.11317400	-0.23398000
C	-1.85055000	-1.94338300	0.45111200
C	-2.88669800	-2.64256600	-0.16059400
C	-3.09015800	-2.51096900	-1.52540400
C	-2.25393400	-1.67461700	-2.25455100
C	-1.23311800	-0.99867300	-1.60184000
C	1.69018800	-0.21815200	-0.24722500
C	2.69971300	0.62079700	0.22614400
C	4.00774800	0.62081700	-0.23504000
C	4.36996900	-0.27035500	-1.23606100
C	3.41454600	-1.14196100	-1.73625600
C	2.11504700	-1.10491900	-1.23721400
C	-0.55185900	1.35003700	0.12783700
C	-1.83661800	1.57383400	0.63004400
C	-2.61720100	2.67657000	0.32401800
C	-2.12209300	3.62758400	-0.56084900
C	-0.86243100	3.44566300	-1.10908700
C	-0.10856400	2.32372900	-0.76655400
C	0.82671100	-1.72956500	2.33464800
C	1.18305600	-2.74684000	1.28719500
H	0.61476000	-2.67577400	0.35974500
H	1.03849100	-3.75014600	1.70553300
H	2.24862500	-2.63490200	1.03456700
C	0.95945100	-2.15414700	3.65192600
H	1.24189100	-3.18950100	3.83543100
C	0.73208400	-1.28180000	4.69932200
H	0.81672300	-1.60867900	5.73634500
C	0.43085000	0.02855500	4.38502300
H	0.29398600	0.77133600	5.16968800
C	0.31199600	0.44607500	3.05951500
C	0.07115200	1.92326700	2.89100800
H	0.51152700	2.33729000	1.98628400
H	0.51931800	2.43220300	3.75381000
H	-1.00503600	2.14076300	2.90239200

==

$Me_3P–B(C_6F_5)_3$

==

B	0.00017771	-0.00082777	0.69926292
P	0.00211430	0.00212612	4.62630847
C	1.28420017	2.87780933	6.04808172
H	1.59600542	2.55395260	8.07610935
H	3.07862512	3.36186488	5.13288168
H	-0.06331016	4.43311538	5.80394800
C	1.85318523	-2.54081212	6.05509071
H	3.87334439	-2.15005519	5.80935072
H	1.41726118	-2.64178004	8.08354164
H	1.37707919	-4.34067362	5.14654251
C	-3.12517194	-0.32445002	6.05764184
H	-2.99346536	0.11087682	8.08449973
H	-3.79761107	-2.27000086	5.81922265
H	-4.44578418	0.98525181	5.14539167
C	-2.77559897	-1.09725921	-0.12350683
C	2.33767320	-1.85594843	-0.12577639
C	0.43729856	2.95103030	-0.12603528
C	-1.12028134	4.85634686	0.81266161
C	-0.90006652	7.39632233	0.22126425
C	0.97050856	8.14385527	-1.46288424
C	2.54385816	6.32649617	-2.50651007
C	2.24965764	3.79447252	-1.84434625
F	3.81211083	2.18293531	-3.01201559
F	4.32365717	7.01032222	-4.14754257
F	1.23685004	10.56473484	-2.07217163
F	-2.45051869	9.10013642	1.23738511
F	-3.01769380	4.23604570	2.41361113
C	-3.65168032	-3.39513622	0.82005610
C	-5.96177609	-4.47300684	0.22686918
C	-7.53950725	-3.22930113	-1.46383855
C	-6.74776427	-0.96140990	-2.51126840
C	-4.40810061	0.04867819	-1.84696730
F	-3.78980291	2.20422916	-3.01869955
F	-8.22511283	0.23591605	-4.15816472
F	-9.76888673	-4.20830404	-2.07549188
F	-6.66720256	-6.66411676	1.24705106
F	-2.16980366	-4.72604822	2.42703385
C	4.76718234	-1.46043298	0.81172808
C	6.85609189	-2.92195781	0.21980350
C	6.56667822	-4.91622714	-1.46360801
C	4.20544294	-5.36957446	-2.50583921
C	2.16065822	-3.84762180	-1.84365461
F	-0.01633548	-4.39486462	-3.01123513
F	3.90611652	-7.25312223	-4.14628401
F	8.52911720	-6.35818891	-2.07365885
F	9.10758786	-2.43155207	1.23447871
F	5.17995321	0.49281694	2.41279288

==

$tBu_3P–B(C_6F_5)_3$

==

C	0.27456400	4.39671500	-1.27247700
C	-0.69386400	3.74739800	-0.51717100
C	-0.88603200	2.38519600	-0.68979300
C	-0.11381400	1.60956800	-1.55849200

C	0.83478200	2.30979500	-2.31088300
C	1.03541500	3.67698300	-2.18853200
B	-0.23919800	0.04354900	-1.60064800
C	-1.64284400	-0.63446000	-1.39971900
C	-1.79330600	-1.81383600	-0.66587400
C	-3.02924700	-2.35533700	-0.34753700
C	-4.18198900	-1.73527000	-0.81307300
C	-4.08304400	-0.58451100	-1.58889800
C	-2.82970900	-0.05529800	-1.86030100
F	-3.11552300	-3.44588900	0.41002700
F	-0.72537200	-2.46450700	-0.19628700
F	-5.37148700	-2.24147100	-0.52666200
F	-5.18468600	-0.00145800	-2.05134800
F	-2.78929300	1.05470400	-2.60164500
F	-1.84063800	1.82439300	0.05772600
F	1.59483100	1.66372700	-3.19848700
F	-1.41388800	4.42531700	0.37310200
F	1.94758100	4.30186800	-2.92666500
F	0.46810700	5.69857000	-1.12810900
C	1.04378400	-0.84499100	-1.78581900
C	1.01294100	-2.08224800	-2.43836200
C	2.11005900	-2.92876800	-2.50535600
C	3.29940400	-2.54998100	-1.89079400
C	3.38371300	-1.32150900	-1.24789100
C	2.27476500	-0.48984300	-1.22747000
F	4.34993100	-3.35499100	-1.92680000
F	2.03454000	-4.09488700	-3.13918100
F	4.51232800	-0.95953700	-0.64303800
F	-0.10561400	-2.49510600	-3.03934100
F	2.42796600	0.68061000	-0.60279000
C	2.03372300	-2.24641300	1.90765300
C	1.02927900	-1.78720900	2.98598400
P	0.36648800	-0.06823100	2.40240400
C	1.65254000	1.25648700	2.97282800
C	1.36450000	2.52121800	2.13634700
C	-0.09646000	-2.83837900	2.97287600
C	1.69777400	-1.84146700	4.36792700
C	-1.26547600	0.27055300	3.38002400
C	-1.63655700	1.76374800	3.31373400
C	-1.29454000	-0.15024100	4.85739200
C	-2.38245200	-0.46624100	2.61106200
C	3.08089200	0.82706400	2.58895400
C	1.66097800	1.61882800	4.46543600
H	0.35867100	-3.83404600	3.10866500
H	1.98588600	-2.88367800	4.59014600
H	2.42204800	-3.24460600	2.17312600
H	-0.81928600	-2.70446600	3.78719800
H	-0.63924600	-2.85843700	2.02044200
H	1.02414500	-1.50948500	5.16911400
H	-1.18256500	-1.23295200	4.99464800
H	2.61257700	-1.23831100	4.42230000
H	1.53914300	-2.32727300	0.92836800
H	-2.24635300	-1.55363300	2.57537500
H	2.89694100	-1.57869600	1.80006700
H	-2.26909000	0.13040400	5.29342900
H	-0.51470900	0.34918300	5.44781800
H	-3.35182600	-0.27217000	3.10138000
H	3.46234700	0.00197900	3.20317300
H	-2.44830700	-0.09552400	1.57771800
H	1.86041000	0.74750600	5.10311200
H	3.16286100	0.54545000	1.53238700
H	3.75559400	1.68492000	2.74938300
H	0.72000000	2.07463700	4.79763200
H	2.46018000	2.35647600	4.65433800
H	-2.66398700	1.88301500	3.69721500
H	-0.98888700	2.39574600	3.93399000
H	-1.62842800	2.15231800	2.28824700
H	1.45122200	2.30093800	1.06222800
H	0.37245800	2.95140600	2.31831600
H	2.10915700	3.29761100	2.38220600

======================================

$Mes_3P–BPh_3$

======================================

C	2.07950200	4.54782800	0.40415200
C	2.79891200	3.63388100	1.16903500
C	2.62646100	2.24922400	1.05238700
C	1.66777900	1.75278700	0.14139200
C	0.96236900	2.67094900	-0.67848900
C	1.17874000	4.04030100	-0.53149800
P	1.07443000	0.00833200	0.01103200
C	1.70674600	-0.75031900	-1.55005400
C	1.01414800	-1.92207200	-1.95011600
C	1.26192000	-2.48070300	-3.20321600
C	2.18200400	-1.92277100	-4.08997700
C	2.88778200	-0.80024500	-3.66593900
C	2.68408500	-0.20749100	-2.41386700
C	0.05686800	-2.63054300	-1.02892800
C	3.58016300	0.95281300	-2.05391800
C	2.40195300	-2.52365700	-5.45373500
C	3.53353200	1.37519200	1.88410600
C	0.02522900	2.20775700	-1.76197400
C	2.26521600	6.03353100	0.56985100
C	1.67355900	-0.96408000	1.46297000
C	2.65317300	-1.98188800	1.44708400
C	2.82939800	-2.77248000	2.58916200
C	2.09364300	-2.58259200	3.75552700
C	1.17148400	-1.53682900	3.77480500
C	0.95061800	-0.72834500	2.66072800
C	3.57838400	-2.24683700	0.28414200
C	-0.01204100	0.42184600	2.79235400
C	2.28434800	-3.46604400	4.96066700
H	3.58825900	-3.55971900	2.56431900
H	0.59892000	-1.34016100	4.68570900
H	0.71297000	-3.38145900	-3.49221800
H	3.64471900	-0.36988500	-4.32780500
H	3.54169300	4.00712800	1.87979200
H	0.62037500	4.72983700	-1.17105900
H	0.59832700	-3.15268400	-0.22237200
H	-0.62224200	-1.91580800	-0.54532200
H	-0.54918700	-3.36607900	-1.57491500
H	3.06355300	1.92199300	-2.11404300
H	3.96545100	0.86766500	-1.02860800
H	4.43995300	0.98724800	-2.73757000
H	2.53199200	-3.61486600	-5.39275200
H	1.53686100	-2.33457900	-6.10990000
H	3.29064300	-2.10054200	-5.94288900
H	0.58408900	1.77927700	-2.61077100
H	-0.64284300	1.41937600	-1.39057800
H	-0.59295700	3.03596400	-2.13391600
H	3.02094400	0.94169200	2.75551400
H	3.93032500	0.53101600	1.30406200
H	4.38490400	1.96393400	2.25337600
H	2.39335000	6.53075000	-0.40366000
H	1.38534500	6.48714000	1.05433200
H	3.14331600	6.26407400	1.18940900
H	0.52669400	1.38186700	2.85821100
H	-0.66873200	0.48497100	1.91439900
H	-0.64123500	0.31326000	3.68603300
H	3.96006600	-1.31474100	-0.15404600
H	4.43892100	-2.84259800	0.61924300
H	3.08600200	-2.79676700	-0.53151800
H	2.39825000	-2.86963600	5.87867700
H	1.41284000	-4.12481400	5.10595300
H	3.17235100	-4.10525500	4.85625000
C	-2.96701600	-2.91539800	-3.34036800
C	-2.35116500	-1.67099100	-3.46569700
C	-2.44272400	-0.74442000	-2.43049100
C	-3.11806600	-1.04076300	-1.23082900
C	-3.72404100	-2.30791700	-1.13056500
C	-3.66333000	-3.22969700	-2.17283100
B	-3.14730300	-0.02236400	-0.03425200
C	-3.14964000	1.52352100	-0.31773200
C	-2.50371100	2.42954700	0.54553000
C	-2.44104000	3.79118500	0.26161200
C	-3.05648800	4.29059600	-0.88528400
C	-3.72339300	3.42086800	-1.74853800
C	-3.75579700	2.05627400	-1.47176900
C	-3.15439000	-0.54973600	1.44608200
C	-3.78938400	0.16869900	2.47737300
C	-3.76170100	-0.27304000	3.79779600

C	-3.07065000	-1.43942000	4.12735400
C	-2.42641600	-2.16814600	3.12852700
C	-2.48451700	-1.73482300	1.80669400
H	-4.26884300	1.38509400	-2.16453200
H	-4.21261500	3.80782800	-2.64472600
H	-3.01694000	5.35925500	-1.10753400
H	-1.90516200	4.46238400	0.93508400
H	-2.01319000	2.05745700	1.44633200
H	-4.32140700	1.09205700	2.23632100
H	-4.27315800	0.29798000	4.57536700
H	-3.03440600	-1.78001800	5.16445400
H	-1.87130700	-3.07378400	3.37950000
H	-1.97065700	-2.31672500	1.03993300
H	-1.95195000	0.22322500	-2.54572500
H	-1.79231700	-1.42242600	-4.36972600
H	-2.90480700	-3.64234300	-4.15319700
H	-4.15285100	-4.20053100	-2.07235100
H	-4.25927900	-2.57353000	-0.21583900

==

$Me_3P–BF_3$

==

C	-1.43742800	-1.34352700	1.02115700
H	-1.06298500	-1.22249300	2.04738000
C	-1.44141800	1.55543800	0.65208700
H	-2.53657500	-1.34310200	1.02052200
H	-1.06268700	-2.29966200	0.62978500
H	-1.06779000	1.69627900	1.67609400
H	-1.06888900	2.38423100	0.03392300
H	-2.54057000	1.55181100	0.65126800
C	-1.43919600	-0.21380800	-1.67339300
H	-1.06916400	0.60535000	-2.30579600
H	-1.06154700	-1.16152100	-2.08208500
H	-2.53834700	-0.21812700	-1.67047300
P	-0.75160300	0.00039200	0.00011600
B	1.30332800	0.00066700	-0.00005100
F	1.65381900	-1.23705400	-0.52160300
F	1.65466700	0.16730900	1.33227300
F	1.65531600	1.07078800	-0.81080300

==

carb···H_2···$B(C_6F_5)_3$

==

B	1.11862800	-0.12824800	0.50172400
N	-3.39908100	-0.74018300	-1.34143000
N	-3.08442000	1.37343700	-1.47054900
C	-4.52797300	-0.14033100	-0.80440800
C	-4.32367100	1.19447300	-0.87492300
C	-2.48952400	0.18667200	-1.76067800
C	-3.30445500	-2.18313800	-1.68367500
C	2.40999800	-0.74107300	-0.18709700
C	1.08538800	1.43976100	0.73568700
C	0.07731000	-1.06612500	1.23991500
C	3.68029000	-0.20121300	0.02586700
C	2.35951300	-1.84937900	-1.03500800
C	3.47873900	-2.37708300	-1.66100400
C	4.72280000	-1.80291000	-1.42358200
C	4.82719400	-0.71243400	-0.56978000
C	0.72416400	2.01668800	1.95329700
C	1.43252300	2.33301900	-0.27944300
C	1.36517700	3.71022400	-0.13010500
C	0.98470900	4.24115000	1.09607500
C	0.67256900	3.39114700	2.15001900
C	0.38892200	-2.35772900	1.67351500
C	-1.24732500	-0.66672200	1.44850200
C	-2.20883900	-1.49277500	2.00814600
C	-1.85916700	-2.78163700	2.39166400
C	-0.55004900	-3.21654400	2.23403500
C	-4.16105600	-3.00687600	-0.71660400
C	-3.82149900	-2.35649500	-3.11780200
C	-1.84851600	-2.63406900	-1.59459400
C	-2.50368100	2.71268700	-1.73402900
C	-1.25668400	2.57333600	-2.60452300
C	-2.14610900	3.36436600	-0.39374900
C	-3.54225200	3.55884000	-2.48224100
H	-4.95546400	2.01026100	-0.54232900
H	-5.37095600	-0.68869200	-0.40277800
H	-5.23716700	-2.82974400	-0.86035800
H	-3.98184900	-4.07553400	-0.90097900
H	-3.91296800	-2.79514600	0.33239400
H	-3.19691300	-1.78032600	-3.81568800
H	-3.79332200	-3.41710500	-3.41081200
H	-4.85938100	-1.99991200	-3.20549700
H	-1.77676600	-3.69641000	-1.87015900
H	-1.20995300	-2.05376300	-2.27039600
H	-1.49737400	2.10759700	-3.57011800
H	-0.83593500	3.57371500	-2.78582200
H	-0.48745700	1.96057400	-2.12500500
H	-1.71112600	4.36205000	-0.55886800
H	-3.03374700	3.48085600	0.24635600
H	-1.41729700	2.74630100	0.14587900
H	-4.44051600	3.75012600	-1.87699100
H	-3.10366300	4.53396400	-2.73967700
H	-3.84998000	3.05941700	-3.41281000
H	-1.45848800	-2.51742200	-0.57493500
H	-0.58058500	-0.01416300	-1.38240200
H	0.18835100	-0.09343100	-1.15495400
F	1.81400400	1.87237600	-1.47605700
F	1.63963000	4.52188400	-1.14935200
F	0.90587200	5.55641200	1.25446500
F	0.32057600	3.89793500	3.32883000
F	0.41941500	1.24457100	3.00176500
F	1.19015000	-2.44679500	-1.28336100
F	3.37550700	-3.42561200	-2.47378100
F	5.80618500	-2.30009500	-2.00520500
F	6.01778200	-0.16958200	-0.32837000
F	3.84812800	0.83789700	0.85138000
F	1.63401500	-2.83032900	1.57551900
F	-0.20628100	-4.44218400	2.61953400
F	-2.77956100	-3.59213800	2.89813000
F	-3.46697700	-1.07974700	2.15562100
F	-1.65145200	0.55210500	1.08536300

==

lut···H_2···$B(C_6F_5)_3$

==

C	-5.07131300	0.72799000	-2.37443600
C	-4.07222700	1.66783200	-2.59950200
C	-2.73829800	1.28303400	-2.45002200
N	-2.40345400	0.03008500	-2.10990800
F	-1.88897900	1.38691800	0.43000500
C	-1.87108200	0.16108100	0.96431200
C	-0.69362900	-0.59272100	0.95677100
C	-0.77608000	-1.83605500	1.58965200
C	-1.95287100	-2.32137900	2.14949200
C	-3.10836300	-1.55307700	2.08620200
C	-3.06898000	-0.29809800	1.49060400
B	0.61828500	-0.06096000	0.23080100
C	0.98393700	1.48764000	0.32220800
C	0.75516800	2.23907700	1.47339200
C	1.09042200	3.58341700	1.57310300
C	1.67741300	4.22193300	0.48688200
C	1.93159600	3.50908700	-0.67892400
C	1.59776100	2.16261100	-0.73200100
F	0.29633400	-2.62478100	1.69740500
F	-1.98199900	-3.51387000	2.73851400
F	-4.16957300	0.44603900	1.43104600
C	1.80177400	-1.07026600	-0.11904700
C	1.58823400	-2.24118700	-0.84747800
C	2.60378300	-3.12550100	-1.17640600
C	3.90013300	-2.85624800	-0.74931200
C	4.15801100	-1.71252400	-0.00526200
C	3.11417300	-0.84310300	0.29397400
F	0.35291200	-2.54665300	-1.26581500
F	3.41929500	0.23117200	1.03004900
F	5.39423500	-1.45865100	0.41531400
F	2.35247500	-4.22366800	-1.88437200
H	-4.31708200	2.69379800	-2.87672000
H	-6.12278400	1.00391500	-2.47257200
H	-0.55724400	-0.08988900	-1.53684800
H	0.22245800	0.02371500	-1.45995400
C	-4.71435600	-0.56970900	-2.02557500
H	-5.47296200	-1.33204300	-1.84471200

C	-3.35985100	-0.88794100	-1.90907000
C	-2.90168800	-2.27825800	-1.57030700
H	-3.73354800	-2.90599800	-1.22397600
H	-2.45346400	-2.75359900	-2.45637600
H	-2.12207400	-2.25200100	-0.79829300
C	-1.60927800	2.25945900	-2.63832500
H	-1.95872500	3.19337400	-3.09771800
H	-1.15833900	2.50155700	-1.66379600
H	-0.82019900	1.82608200	-3.26844200
F	4.88465900	-3.69386300	-1.04753000
F	-4.24216500	-2.01135000	2.59949200
F	2.49475900	4.11467400	-1.72134300
F	0.85741600	4.26195500	2.69331300
F	1.99812700	5.50736900	0.56396000
F	0.20782400	1.66797100	2.55222000
F	1.87361400	1.51037300	-1.87123300

==

$Me_3P\cdots H_2\cdots B(C_6F_5)_3$

==

C	4.69673848	-1.95978221	2.25784292
C	2.94432362	-2.08278250	0.29515824
C	3.25813900	-4.07296649	-1.40133017
C	5.17250329	-5.85512762	-1.15733062
C	6.85260967	-5.67784116	0.85245548
C	6.61638078	-3.71502608	2.58412114
B	0.79541976	0.00768746	-0.04282117
C	-1.81195070	-0.86046771	-1.26655301
C	-2.98156639	0.41269564	-3.25463750
C	-5.20953464	-0.43045658	-4.36949466
C	-6.35838998	-2.61282753	-3.47200325
C	-5.25949351	-3.93683397	-1.48574801
C	-3.02342400	-3.05628785	-0.44251903
F	-1.96097433	2.50832628	-4.22528588
F	-6.24740997	0.83804104	-6.27519654
F	-8.49818361	-3.42472936	-4.49388210
F	-6.37559422	-6.00846697	-0.58538044
F	-2.03733473	-4.39854714	1.45918418
F	1.72580542	-4.31215472	-3.39840408
F	5.41125876	-7.71810847	-2.82817166
F	8.68233502	-7.37176530	1.11604582
F	8.22390089	-3.53375796	4.50821006
F	4.53396134	-0.07993367	3.95324096
C	1.66523628	2.88612393	-0.26915934
C	3.97438150	3.57842120	-1.33463417
C	4.74146304	6.08244624	-1.56496344
C	3.16754047	7.99860783	-0.70643818
C	0.83990051	7.39560956	0.35460902
C	0.13778020	4.87802196	0.53221682
F	5.55394868	1.82361765	-2.23469750
F	6.95916629	6.65229304	-2.60085651
F	3.87509185	10.39526052	-0.90352150
F	-0.68207537	9.22219058	1.17461978
F	-2.13510494	4.38697674	1.54752510
P	-5.47940829	0.20576131	4.46011086
H	0.35255867	0.16116726	3.13472876
H	-1.14562566	0.08747179	3.19693476
C	-7.66974253	1.73142236	2.20908232
H	-7.82092635	0.56898947	0.49302768
H	-9.56934814	1.97870824	3.02400781
H	-6.90712078	3.58424778	1.66170047
C	-5.95865785	2.21075320	7.27769063
H	-4.98299985	1.36701500	8.90822760
H	-5.12337046	4.08194297	6.92489388
H	-7.97581199	2.44184602	7.73740998
C	-7.28916514	-2.65178419	5.32776160
H	-7.28652148	-3.98560392	3.73434075
H	-6.35996228	-3.58784874	6.93519478
H	-9.25639837	-2.20748113	5.84434848

==

$tBu_3P\cdots H_2\cdots B(C_6F_5)_3$

==

H	3.38790900	-3.70821900	-1.89679600
H	4.40400800	-2.48806500	-3.73539900
H	1.94719900	-2.92193000	-3.50750400
F	1.45573700	-3.95281100	1.43140900
C	3.35254300	-2.78276100	-1.29839600
H	4.30279500	-2.71534900	-0.75438000
H	2.53772500	-2.90157500	-0.57141000
F	-5.40287900	-2.66595600	-2.57765800
C	4.27551100	-1.50997300	-3.24046600
H	5.22656300	-1.25991000	-2.75229700
F	-4.84807800	-3.49913200	-0.04990100
C	1.81401500	-1.94529700	-3.01232200
H	5.63296600	-1.26704800	-0.57681500
C	3.10862100	-1.59622000	-2.24713300
F	-0.10187600	-2.49086400	-0.17750200
H	4.09480500	-0.77068000	-4.03235600
C	0.88934300	-2.83857000	1.89383600
H	0.95979400	-2.03239100	-2.32656200
F	1.89265200	-3.16812400	3.99637800
C	-4.47581800	-2.04307900	-1.86207200
C	-4.18765600	-2.46914000	-0.57175000
H	3.60807700	-1.82178400	1.05747300
H	1.55918700	-1.21928100	-3.79147200
C	0.09154200	-2.05460000	1.07407500
H	6.21861200	-0.04482100	0.56058500
C	1.12078400	-2.43730900	3.20317200
F	-4.06743900	-0.54859100	-3.63776600
C	5.52970700	-0.21435400	-0.28468800
C	-3.79239000	-0.95844500	-2.40240300
F	-2.98276400	-2.25059000	1.40276500
C	-3.20789100	-1.80890000	0.16054700
H	5.87640800	0.40755800	-1.12157000
C	3.65891300	-0.75760000	1.31714400
H	4.37768100	-0.65600200	2.14785500
H	2.53658900	0.54402400	-4.32865200
C	-2.83408100	-0.31510200	-1.63377200
C	-2.49619800	-0.71973300	-0.34134600
P	2.73980100	-0.02063400	-1.20535400
C	4.10146000	0.14645200	0.14845400
C	-0.48789500	-0.85671600	1.49473100
H	2.66951000	-0.44865000	1.68687300
C	0.54448800	-1.26439300	3.67471100
H	4.82676200	1.14817100	-3.26977100
H	0.44524500	0.04092800	-0.83126200
H	-0.33348200	0.12766600	-0.95279900
F	-2.20143700	0.72858600	-2.18849700
C	-0.24133100	-0.49999600	2.82062300
B	-1.37976300	0.01733000	0.51525600
F	0.75056900	-0.88309500	4.93244000
C	2.04485900	1.25006800	-3.64741400
H	1.03029500	0.88338800	-3.42958100
C	4.26900000	1.95090100	-2.76899000
C	2.85484900	1.50954200	-2.36489400
H	1.93813900	2.20366200	-4.19084000
H	4.86430300	2.29340700	-1.91299900
C	4.12969000	1.57406500	0.72465900
H	4.19983700	2.79634500	-3.47510900
F	-0.78395400	0.60893400	3.33427000
H	4.79998600	1.57701800	1.60039600
H	4.52264500	2.31591600	0.01838800
H	3.14148700	1.90438900	1.06839300
C	-1.43300200	1.59432100	0.68434500
F	0.88273500	1.66949000	1.14944100
F	-3.76836100	1.76599400	0.29233700
H	1.06670300	2.42354900	-1.49229400
C	2.12745400	2.66992600	-1.65365000
H	2.56247300	2.94347900	-0.68726300
C	-0.27419500	2.31899100	0.96864000
C	-2.59311900	2.35445700	0.53081500
H	2.16334200	3.56390700	-2.29910600
C	-0.23512100	3.70036900	1.05375000
C	-2.60091600	3.74248200	0.62859200
F	0.91128300	4.33591000	1.28872200
F	-3.73175400	4.42576400	0.47644600
C	-1.41386700	4.41860700	0.88165200
F	-1.40460700	5.74194800	0.96240500

==

$Mes_3P\cdots H_2\cdots BPh_3$

==

C	-3.52088600	-1.37066600	-0.56891500
C	-3.32995000	-0.74246500	0.67376700
C	-3.96994200	-1.32742400	1.77707400
C	-4.73104800	-2.49252600	1.65673900
C	-4.87786000	-3.10876300	0.41635300
C	-4.27150100	-2.53518000	-0.70276400
B	-2.48907200	0.63286700	0.78450400
C	-1.79176700	0.98419400	2.19735300
C	-1.55872900	2.30878000	2.61012300
C	-0.92199400	2.61088200	3.81461900
C	-0.48156000	1.58603200	4.64997900
C	-0.67512200	0.26164200	4.26047600
C	-1.31940100	-0.02518600	3.05779000
C	-3.23315900	1.82210700	-0.01197700
C	-4.13566500	2.66502700	0.65943000
C	-4.87341400	3.64076200	-0.01047600
C	-4.73396900	3.80337600	-1.38784000
C	-3.86074300	2.96939700	-2.08415700
C	-3.13264800	1.99592800	-1.40149400
C	-0.94746900	-3.18290500	-2.05970200
C	-0.81198000	-3.05386500	-3.43979800
C	-0.01412200	-2.01801600	-3.91872500
C	0.65694500	-1.12795400	-3.07339700
C	0.53014300	-1.29849400	-1.67669500
C	-0.30805500	-2.32457400	-1.16650300
P	1.17832900	-0.14509900	-0.40036600
C	2.21624700	-1.01371500	0.84963300
C	2.76170400	-2.31103000	0.72937600
C	3.42650900	-2.86135100	1.83216100
C	3.58785000	-2.17800700	3.03243900
C	3.09361200	-0.87590600	3.10801800
C	2.42101100	-0.27650300	2.04539400
C	2.72956300	-3.17860800	-0.50974500
C	4.27410300	-2.81154400	4.21320000
C	1.98328200	1.15617500	2.20274200
C	1.46209600	-0.03356200	-3.73375300
C	-1.53009500	-3.99326600	-4.37177000
C	-0.54286200	-2.56725800	0.30547100
C	2.21764800	1.24018400	-1.03736600
C	1.61434700	2.51515000	-1.11244900
C	2.39261000	3.61852000	-1.47283300
C	3.75117400	3.50574100	-1.75272300
C	4.32637200	2.23682100	-1.68299500
C	3.59449200	1.10212100	-1.33291900
C	0.15365400	2.74138000	-0.83002400
C	4.32657700	-0.21457000	-1.32182900
C	4.58331600	4.70693600	-2.11434700
H	5.20485800	4.50703600	-3.00003500
H	-1.42448600	0.35922200	-0.07472100
H	5.38746100	2.12061900	-1.92015600
H	-0.56617800	-1.64279300	0.89499500
H	-0.48786900	2.09364700	-1.44133100
H	5.25828300	-0.13577200	-1.89887600
H	-0.09901000	2.54252700	0.22288900
H	1.91246600	4.59820600	-1.53434400
H	4.58591300	-0.52464700	-0.29878600
H	-1.59709700	-3.96226600	-1.65338800
H	-0.13307400	3.77824300	-1.04795600
H	0.08751100	-1.88327900	-4.99874900
H	0.92310200	1.29571700	1.94708000
H	3.23571900	-0.30003400	4.02610700
H	4.99070000	-2.11762900	4.67716400
H	3.72819600	-1.02166800	-1.76392100
H	2.11034400	1.48594500	3.24069000
H	2.57460600	1.82285700	1.55610400
H	-1.19928600	-5.03187300	-4.21575500
H	-2.61611200	-3.96676500	-4.19266300
H	-1.35221300	-3.73402200	-5.42456400
H	0.24391400	-3.20018800	0.74283800
H	1.37814300	-0.11782000	-4.82563200
H	1.11426100	0.96902300	-3.44835400
H	2.52883700	-0.08633200	-3.47790700
H	-1.50961600	-3.06575400	0.45347800
H	3.84322600	-3.86770400	1.73405300
H	3.53890100	-3.08730900	4.98606600
H	4.81522800	-3.72349500	3.92527600
H	2.54471400	-2.62786400	-1.43535900
H	3.69055400	-3.70348700	-0.61008500
H	-0.47937700	0.30452400	0.03452200
H	1.94347900	-3.94670400	-0.44041400
H	3.95626400	5.58412000	-2.32473600
H	5.26445400	4.96958600	-1.28924600
H	-2.46856800	1.34165300	-1.97562900
H	-3.74904600	3.07423100	-3.16600400
H	-5.30700600	4.56869900	-1.91551100
H	-5.56451100	4.27703800	0.54751800
H	-4.27282300	2.54954600	1.73823600
H	-1.89168700	3.13171000	1.97312600
H	-0.76998200	3.65394200	4.10178600
H	0.01402300	1.81777600	5.59539100
H	-0.32558000	-0.55430500	4.89713700
H	-1.47130000	-1.07275800	2.78781600
H	-3.87595800	-0.86147000	2.76121500
H	-5.21265300	-2.91971900	2.53959600
H	-5.46851500	-4.02227100	0.31884900
H	-4.38698100	-2.99762500	-1.68641600
H	-3.05168800	-0.94505200	-1.46007200

==

$Me_3P\cdots H_2\cdots BF_3$

==

P	1.25411500	-0.00954400	-0.22735900
B	-1.98696100	-0.01087600	-0.24004800
C	2.74681000	-0.69436100	-1.04226800
H	2.93909100	-0.15204300	-1.97914100
H	-0.29331700	-0.00751600	-1.04389900
F	-3.23171300	0.29266100	-0.70960100
H	-1.20856400	0.06231500	-1.39592200
C	1.70905100	1.69361700	0.25829500
H	3.62867500	-0.61218800	-0.38893800
H	2.57466300	-1.75340300	-1.28269500
H	1.93187100	2.28787600	-0.63951800
H	0.83884000	2.13281200	0.76535900
H	2.58131700	1.70292800	0.92869900
C	1.10671800	-0.92289600	1.34657900
H	0.25929300	-0.49572500	1.89897200
H	0.87634400	-1.97546400	1.13269400
H	2.03089700	-0.85023600	1.93853000
F	-1.81565200	-1.29941200	0.22555800
F	-1.44280200	0.94008800	0.62858100

==

$carbH\cdots HB(C_6F_5)_3$

==

B	-0.69174600	-0.13843000	-0.11554400
N	3.51819500	0.60879800	0.98552900
N	2.21059000	2.11306200	1.86738200
C	3.99951500	1.84433800	0.60617700
C	3.17993800	2.78006900	1.15152200
C	2.42838600	0.80497600	1.73630200
C	4.19787800	-0.71535500	0.79021700
C	-1.28399900	-1.50727200	0.54670200
C	-1.88464000	0.90629100	-0.50287000
C	0.38144900	-0.38714500	-1.32456800
C	-2.39885200	-2.17567600	0.04263200
C	-0.74000000	-2.07749200	1.68997500
C	-1.23487000	-3.22107400	2.30687000
C	-2.34941000	-3.84583800	1.76632600
C	-2.93732900	-3.31787000	0.62249200
C	-2.27458100	1.25720700	-1.79240300
C	-2.58260300	1.55353100	0.51339200
C	-3.57233100	2.50296400	0.29413900
C	-3.91792800	2.82748600	-1.01118900
C	-3.26593500	2.19629400	-2.06242700
C	0.53335500	-1.54595400	-2.08318400
C	1.32339500	0.60312200	-1.59048100
C	2.37888700	0.45516000	-2.47836400
C	2.50989500	-0.73238500	-3.18519500
C	1.56932000	-1.73606800	-2.99700100
C	5.17950900	-0.59778300	-0.37627000
C	4.94489800	-1.03357300	2.09031800
C	3.14678200	-1.78150600	0.48782800

C	1.07608200	2.76472500	2.60253800
C	0.34730800	1.70422900	3.42484000
C	0.14583600	3.39420100	1.56288000
C	1.67642500	3.82364000	3.53168900
H	3.19821500	3.85875700	1.05974700
H	4.85810700	1.96577500	-0.04013300
H	6.02143800	0.07091500	-0.14345800
H	5.60092100	-1.59217500	-0.57587900
H	4.68699100	-0.24894800	-1.29374200
H	4.24374300	-1.14670500	2.93066400
H	5.49387000	-1.97933800	1.97740800
H	5.66729200	-0.24120200	2.33742300
H	3.65295300	-2.74860700	0.36156500
H	2.41192500	-1.89456400	1.29536200
H	1.01953300	1.23304700	4.15847700
H	-0.46602900	2.19420400	3.97607900
H	-0.11020200	0.93718400	2.78837200
H	-0.74011000	3.80458400	2.06741300
H	0.64582900	4.21318400	1.02381200
H	-0.18160600	2.64497600	0.83292300
H	2.19464600	4.62031900	2.97797400
H	0.86297900	4.29633900	4.09965400
H	2.38152800	3.37385400	4.24665400
H	2.60751700	-1.54905200	-0.43726400
H	1.80180500	0.02176800	2.14734700
H	-0.04381500	0.41260100	0.76121700
F	-2.29472100	1.28069000	1.80435900
F	-4.18125500	3.10948000	1.31881600
F	-4.86488800	3.73502400	-1.25171500
F	-3.59718700	2.49733300	-3.32016100
F	-1.69680500	0.68353600	-2.85887400
F	0.35467500	-1.52689600	2.29213900
F	-0.65393900	-3.71600500	3.40480900
F	-2.84809700	-4.94285800	2.33564300
F	-4.00486900	-3.91770200	0.09429200
F	-2.99730300	-1.72899900	-1.06874200
F	-0.32091600	-2.56837900	-1.95351900
F	1.67842100	-2.87740400	-3.67833700
F	3.54034900	-0.90718400	-4.01499400
F	3.30197600	1.42044700	-2.62106000
F	1.26138500	1.78478300	-0.94061200

=======================================

lutH···HB$(C_6F_5)_3$

=======================================

C	4.70818900	-1.28190200	-2.11890500
C	3.62645500	-1.60610600	-2.93002100
C	2.48829800	-0.81218700	-2.90009300
N	2.50150700	0.27417200	-2.09856800
F	1.70362800	-1.95740400	-0.36186300
C	1.78299300	-1.11504500	0.69058700
C	0.77837000	-0.17451600	0.90474100
C	0.95719200	0.61665300	2.03910200
C	2.07313300	0.52551700	2.86870200
C	3.07205900	-0.39523700	2.57722300
C	2.92253200	-1.22993400	1.47755800
B	-0.42823300	-0.03372500	-0.19262700
C	-1.51021100	-1.24683600	-0.09469200
C	-1.88761800	-1.87807400	1.08784500
C	-2.79287500	-2.93266700	1.13579400
C	-3.36549700	-3.39576100	-0.04233100
C	-3.02412400	-2.79615100	-1.24728100
C	-2.11483400	-1.74468100	-1.24477300
F	0.04301300	1.53064400	2.38488100
F	2.19940800	1.31803200	3.93312000
F	3.88457600	-2.10867700	1.16846900
C	-1.11762100	1.44847100	-0.22645400
C	-0.59939800	2.46744100	-1.01348500
C	-1.13849200	3.74298300	-1.12183300
C	-2.28421300	4.04282200	-0.39907600
C	-2.84938500	3.06512300	0.41161300
C	-2.26184200	1.80677000	0.48608700
F	0.53677700	2.26341300	-1.75835200
F	-2.84475700	0.92813100	1.30864200
F	-3.94395400	3.34926900	1.11630900
F	-0.57053000	4.66729000	-1.90156800
H	3.64275400	-2.48780200	-3.56856100
H	5.59429700	-1.91810600	-2.11507100
H	1.62377200	0.80399600	-2.00781900
H	0.10842500	-0.16487500	-1.28192300
C	4.66797200	-0.14990700	-1.30902500
H	5.50477700	0.11376700	-0.66459300
C	3.53184800	0.64443100	-1.30526400
C	3.36260700	1.87562600	-0.47852800
H	2.55747400	1.72358900	0.25588000
H	4.29128200	2.10845900	0.05441300
H	3.07860600	2.73109100	-1.10721400
C	1.24145500	-1.09235800	-3.66680600
H	1.41990400	-1.86321200	-4.42526400
H	0.45346800	-1.43114300	-2.97681400
H	0.86527700	-0.18252900	-4.15544100
F	-2.83125500	5.25440100	-0.47623900
F	4.15971800	-0.48009400	3.34319800
F	-3.56561900	-3.23430700	-2.38781200
F	-3.12001600	-3.50199700	2.29768300
F	-4.23501400	-4.40565500	-0.01641900
F	-1.38266500	-1.47109400	2.26199500
F	-1.82346200	-1.20647000	-2.44966400

=======================================

Me_3PH···HB$(C_6F_5)_3$

=======================================

F	-5.81623926	-6.84314413	-0.18874399
F	9.88076715	-5.75367440	1.28618537
F	6.91671278	-6.19101734	-2.89933125
F	-2.02044262	-4.53253462	2.16371182
C	-4.74086593	-4.81862011	-1.26859208
F	-7.53240718	-5.08009003	-4.74179344
C	7.74441104	-4.43080363	1.10590361
C	6.22597639	-4.65100389	-1.02360610
C	-2.76606614	-3.56165376	-0.07699125
C	-5.64543998	-3.91019847	-3.55891748
F	8.47574838	-2.58429369	5.09576365
C	7.02303841	-2.81352306	3.04352296
F	2.64741719	-3.51953017	-3.30330556
C	4.00602636	-3.25294469	-1.17487867
C	4.78511897	-1.45704728	2.81296692
C	3.19344421	-1.62565423	0.72399562
P	-5.67846651	0.07737689	4.22469250
C	-1.63042377	-1.37275948	-1.00285123
C	-4.54342339	-1.76188795	-4.59468393
H	-3.05073620	-0.20918699	3.72647943
H	-0.15419231	0.00906110	2.86890092
F	4.18445071	0.08937801	4.73862053
C	-2.58711836	-0.54755382	-3.31545272
B	0.58873840	0.00498870	0.66137388
F	-5.37824199	-0.88137568	-6.80058392
F	-1.63933556	1.50246450	-4.44981564
C	1.02322817	2.97349332	-0.07256741
F	-2.34079986	4.28274490	2.63634934
F	4.33271684	2.19167187	-2.98523064
C	-0.43289831	4.87563125	0.99473858
C	2.85451268	3.83635162	-1.75947112
C	-0.14854352	7.43699250	0.50202083
C	3.23382243	6.37851513	-2.31107651
F	-1.64696019	9.14542405	1.60873894
F	5.01655608	7.09044101	-3.94103522
C	1.72202272	8.19830069	-1.16974618
F	2.05076150	10.63922779	-1.68720611
C	-7.01386810	-2.93422288	5.14491731
H	-6.73126341	-4.31906145	3.62558317
H	-6.03916818	-3.60809360	6.84936525
H	-9.04082424	-2.71218373	5.53676337
C	-7.26130483	1.26591834	1.44299295
H	-7.10444441	-0.13760961	-0.07785486
H	-9.26026090	1.62694118	1.87463853
H	-6.31290032	3.01881313	0.86954800
C	-6.03713263	2.31482966	6.78635233
H	-5.08589367	1.57543835	8.47681440
H	-5.13956742	4.09765772	6.22035479
H	-8.05087769	2.61844262	7.18977673

=======================================

$tBu_3PH\cdots HB(C_6F_5)_3$

==

H	3.36521200	-4.11055900	-0.62332200
H	4.42240900	-3.45246500	-2.69820700
H	1.82863100	-3.74277100	-2.23214900
F	1.24979400	-3.45543100	2.68895300
C	3.30543000	-3.06633600	-0.27956700
H	4.22620500	-2.86071100	0.27935800
H	2.44483600	-3.00069600	0.39658900
F	-5.46742100	-3.01193700	-2.05137300
C	4.34631000	-2.38052200	-2.45742000
H	5.29500500	-2.07948300	-1.99196700
F	-5.65469000	-1.85893300	0.40876700
C	1.84374300	-2.64238900	-2.24126200
H	5.57129200	-1.43591200	0.15374600
C	3.13720900	-2.18487000	-1.53064000
F	0.22052500	-2.46860300	0.43898100
H	4.23714500	-1.84334500	-3.40901600
C	0.57531700	-2.29702100	2.72890300
H	0.93397600	-2.29703300	-1.73507400
F	0.94507400	-2.10929600	5.05425700
C	-4.45479400	-2.28790600	-1.57379200
C	-4.54656400	-1.69875800	-0.31742700
H	3.39319700	-1.48287700	1.71304100
H	1.79977600	-2.32666300	-3.28809000
C	0.04410000	-1.74614300	1.57055500
H	6.12695900	0.01979400	0.99128000
C	0.43752700	-1.61061600	3.92714000
F	-3.20536900	-2.67972100	-3.53603700
C	5.49824500	-0.34167900	0.16288100
C	-3.30206800	-2.11577900	-2.32752200
F	-3.61469000	-0.38847500	1.36930500
C	-3.47774000	-0.95016100	0.15846100
H	5.93250200	0.04481900	-0.77145300
C	3.45799300	-0.38944800	1.68774300
H	4.09937100	-0.07693700	2.52660300
H	2.61323200	-0.63753400	-4.13769200
C	-2.25930000	-1.35591200	-1.80782800
C	-2.30141700	-0.75036800	-0.55798500
P	2.92824800	-0.36916000	-1.02993000
C	4.06664800	0.17041700	0.38546800
C	-0.60291500	-0.51695000	1.50860900
H	2.45296700	0.02203600	1.86207000
C	-0.23934000	-0.39713800	3.93201100
H	4.88001300	0.26128400	-3.27624200
H	1.61133600	-0.29076500	-0.51101800
H	-0.09798200	-0.15258600	-0.70341400
F	-1.16772400	-1.23330200	-2.59857900
C	-0.74492600	0.11831700	2.74099900
B	-1.04362800	0.07881000	0.04663200
F	-0.39094300	0.26620100	5.07928700
C	2.09222900	0.19885600	-3.65543700
H	1.09161000	-0.12848600	-3.33904500
C	4.31470000	1.15449400	-2.97516600
C	2.89467200	0.81698100	-2.49801500
H	1.95807400	0.98160800	-4.41827600
H	4.89930800	1.69371200	-2.21869300
C	4.12418700	1.70143700	0.53584900
H	4.23841400	1.80970800	-3.85701200
F	-1.37767700	1.29461300	2.83231900
H	4.75234200	1.91505900	1.41483700
H	4.59201900	2.19984600	-0.32241200
H	3.14019800	2.14450700	0.72189900
C	-1.17732300	1.70562500	0.02431400
F	1.02969300	1.81727600	0.87956600
F	-3.37807400	1.87891100	-0.85836900
H	1.08473700	1.85159000	-1.83515500
C	2.13158700	2.08655800	-2.07516600
H	2.57324500	2.61771500	-1.22682600
C	-0.07550200	2.45917500	0.41260000
C	-2.24640700	2.46395200	-0.44462400
H	2.12918900	2.77723300	-2.93286100
C	0.01509800	3.83824700	0.33615600
C	-2.21485600	3.85631200	-0.52922600
F	1.14320900	4.47040300	0.68688100
F	-3.26930400	4.52978000	-0.99119700
C	-1.07677600	4.55144800	-0.14282400
F	-1.02809300	5.88010500	-0.23636400

==

$Mes_3PH\cdots HBPh_3$

==

C	-3.87155200	-1.89517600	-0.14453700
C	-3.24563400	-1.38637100	1.01122600
C	-3.20907700	-2.25563600	2.11443300
C	-3.71367800	-3.55775900	2.05978900
C	-4.29020000	-4.04164800	0.88719500
C	-4.37700000	-3.19216100	-0.21774200
B	-2.53461400	0.08657700	0.95328000
C	-2.06787100	0.69249600	2.39434500
C	-2.50714700	1.91750400	2.92374000
C	-1.97752300	2.46053700	4.09709300
C	-0.97954000	1.78880400	4.80051100
C	-0.52577200	0.56205300	4.31382900
C	-1.06322900	0.03864700	3.13761700
C	-3.41204400	1.09781000	0.03154100
C	-4.69880400	1.53030700	0.40346800
C	-5.47300900	2.35661400	-0.41013700
C	-4.98738200	2.77656500	-1.65001700
C	-3.72482100	2.35186800	-2.05926800
C	-2.96128800	1.52989300	-1.22667900
C	-1.44366600	-1.82689500	-2.47300800
C	-1.59440800	-1.03029400	-3.60679800
C	-0.68338900	0.00407400	-3.81321300
C	0.32475000	0.30768700	-2.89703800
C	0.41361500	-0.48120200	-1.73056700
C	-0.46115600	-1.57799400	-1.51866700
P	1.48797900	-0.07677900	-0.33060400
C	2.66435500	-1.38486100	0.13389700
C	3.13784500	-2.36389600	-0.76849200
C	4.03544700	-3.31704800	-0.28712500
C	4.47653000	-3.33208800	1.03697700
C	4.00647500	-2.34252000	1.89767900
C	3.10663600	-1.36206500	1.47657400
C	2.74842700	-2.45145700	-2.22515500
C	5.42357300	-4.39810100	1.51759300
C	2.66092400	-0.31693600	2.46863000
C	1.23485500	1.46655300	-3.22514400
C	-2.74493800	-1.25246100	-4.54726600
C	-0.37521100	-2.50996100	-0.33611500
C	2.29856700	1.55628800	-0.33504900
C	1.53821600	2.64715000	0.15207700
C	2.14141700	3.90464600	0.19781300
C	3.45735900	4.11465700	-0.20966800
C	4.17892500	3.02177100	-0.68926600
C	3.63176300	1.74055700	-0.76485200
C	0.12381900	2.51265800	0.64128000
C	4.50779700	0.64542400	-1.32023900
C	4.07812300	5.48222500	-0.11750000
H	3.39520800	6.25293700	-0.50356800
H	-1.46324900	-0.09229600	0.34368200
H	5.20691500	3.17034000	-1.02828900
H	-0.28492000	-1.97127400	0.61698900
H	-0.52062900	1.92105600	-0.02376000
H	5.38651200	1.08235500	-1.81237700
H	0.08802300	2.02405100	1.62851100
H	1.55260400	4.74750300	0.56698700
H	4.86436200	-0.03296100	-0.53146500
H	-2.13924100	-2.64823000	-2.29313100
H	-0.34387700	3.49798500	0.75670200
H	-0.77261500	0.61951800	-4.71145700
H	1.57638900	-0.35242000	2.65180100
H	4.35162400	-2.32521200	2.93408800
H	6.19501200	-4.61498700	0.76466800
H	3.98712200	0.03055700	-2.06708700
H	3.15958900	-0.46752500	3.43430200
H	2.89671300	0.70221100	2.12625400
H	-2.55733800	-0.80855300	-5.53472400
H	-2.95968600	-2.32270000	-4.67627300
H	-3.64916500	-0.77932800	-4.13073400
H	0.47971400	-3.19886700	-0.42537000

H	1.07905100	1.77333800	-4.26756500
H	1.03520300	2.33976000	-2.58706700
H	2.29799200	1.21517200	-3.10940500
H	-1.29642300	-3.10110100	-0.25914800
H	4.41051400	-4.07459500	-0.97990100
H	5.92229000	-4.10467500	2.45123900
H	4.87881200	-5.33624300	1.70947700
H	2.70079900	-1.47439700	-2.72251200
H	3.47466000	-3.07083300	-2.76790900
H	0.57062400	-0.01060100	0.74057900
H	1.75589100	-2.90904100	-2.35108500
H	4.29458700	5.73352800	0.93295900
H	5.02042800	5.53968000	-0.67906900
H	-3.96248700	-1.24303900	-1.01834300
H	-4.85284300	-3.54180700	-1.13875700
H	-4.68707000	-5.05842100	0.83966000
H	-3.66088600	-4.19755900	2.94509100
H	-2.77551000	-1.90686400	3.05530300
H	-5.11309400	1.19175500	1.35853000
H	-6.46730600	2.66982700	-0.08071200
H	-5.59276700	3.42063200	-2.29222400
H	-3.33161100	2.65926400	-3.03288100
H	-1.98322900	1.18715900	-1.57799800
H	-0.68540900	-0.92482700	2.77283600
H	0.24989500	0.01232400	4.85536300
H	-0.56316100	2.21145400	5.71760700
H	-2.34765700	3.42172200	4.46426600
H	-3.27343900	2.48147900	2.38598700

==

$Me_3PH\cdots HBF_3$

==

P	1.24687500	-0.01304600	-0.14549400
B	-1.95974300	0.01871800	-0.46622800
C	2.75307100	-0.78586600	-0.80637600
H	3.00652800	-0.33609300	-1.77626500
H	0.22432000	-0.21173200	-1.09211600
F	-3.32081900	0.16891800	-0.28481600
H	-1.57451500	0.26549200	-1.62724500
C	1.54567400	1.75836700	0.04430000
H	3.58980900	-0.64352600	-0.10828200
H	2.57503100	-1.86138700	-0.94555400
H	1.84616800	2.18410100	-0.92264100
H	0.59213000	2.19520800	0.36802500
H	2.33538300	1.92810700	0.78901100
C	0.86173900	-0.78920400	1.43485000
H	0.01080500	-0.24204600	1.85662200
H	0.54555800	-1.82175800	1.24279300
H	1.74836500	-0.75159500	2.08291800
F	-1.52307000	-1.29547600	-0.08646400
F	-1.24132100	0.93739600	0.43901600

==

$^-HB(C_6F_5)_3$

==

B	0.05670300	-0.06082700	0.86910300
C	-1.20000700	-0.98470000	0.37009700
C	-0.20941100	1.47770200	0.35867200
C	1.53585900	-0.63896400	0.47021800
C	-1.62973100	-1.01547400	-0.95439800
C	-1.95562800	-1.75302100	1.25061400
C	-3.06600600	-2.49642800	0.85577900
C	-3.45739900	-2.48775300	-0.47465400
C	-2.73017700	-1.74097600	-1.39272900
C	0.44482800	2.13463900	-0.67967300
C	-1.19820200	2.22968000	0.99251700
C	-1.51465800	3.53865200	0.64585200
C	-0.82401800	4.15351300	-0.38941000
C	0.16318400	3.44455700	-1.05806000
C	1.81802100	-1.81415000	-0.21958500
C	2.66447900	0.04965400	0.91871800
C	3.96976000	-0.37135800	0.70416100
C	4.19697900	-1.55079000	0.00633100
C	3.11167200	-2.27631800	-0.45759800
H	0.03610600	-0.02709800	2.08761900
F	-1.91336700	1.69325700	1.99491000
F	-2.47509000	4.21421400	1.29464700
F	-1.11096400	5.41417500	-0.74173200
F	0.82905200	4.02833100	-2.06534100
F	1.40159200	1.51514500	-1.39094300
F	-1.63852700	-1.82178400	2.55252800
F	-3.76273600	-3.22058800	1.74479700
F	-4.52276100	-3.19638500	-0.87226800
F	-3.09697100	-1.73686300	-2.68263600
F	-0.95137400	-0.33257500	-1.89239700
F	0.83406600	-2.59269900	-0.70579500
F	3.31746800	-3.41767500	-1.13142500
F	5.44756600	-1.97968900	-0.21262700
F	5.01128900	0.33941600	1.16108200
F	2.51554600	1.19821400	1.60117400

==

$^-HBPh_3$

==

C	-1.14324100	-2.37877900	1.03932400
C	-1.07476300	-1.12629000	0.39972300
C	-1.94218700	-0.95034000	-0.69199700
C	-2.81851000	-1.95069000	-1.12205000
C	-2.85891600	-3.17933600	-0.46517700
C	-2.01032100	-3.38833600	0.62453300
B	0.00037400	-0.00018500	0.90030500
C	-0.43773700	1.49399900	0.39995500
C	0.15297400	2.16176800	-0.68645600
C	-0.27610700	3.42091500	-1.11560600
C	-1.32558000	4.06561400	-0.46324500
C	-1.93558500	3.43059600	0.62117800
C	-1.49382000	2.17505100	1.03517700
C	1.51320700	-0.36810100	0.39975800
C	1.79520000	-1.20753100	-0.69159400
C	3.09993800	-1.46584300	-1.12117300
C	4.18379200	-0.88582700	-0.46424200
C	3.93982800	-0.04622400	0.62514500
C	2.63175500	0.19941000	1.03950100
H	0.00039300	-0.00011800	2.14342400
H	-0.48504200	-2.55892800	1.89553500
H	-2.02887600	-4.34601200	1.15431900
H	-3.54410900	-3.96522600	-0.79531300
H	-3.47666200	-1.76755700	-1.97722400
H	-1.93292300	0.00774100	-1.22106200
H	0.96122600	-1.67899200	-1.22085700
H	3.27091300	-2.12760200	-1.97608700
H	5.20713900	-1.08596000	-0.79411200
H	4.77809700	0.41714000	1.15501300
H	2.45819700	0.85975300	1.89542100
H	-1.98284300	1.69148600	1.88712300
H	-2.76023200	3.92176100	1.14736300
H	-1.66435100	5.05187900	-0.79282300
H	0.21530300	3.90288600	-1.96663800
H	0.98234000	1.67833600	-1.21213600

==

$^-HBF_3$

==

B	0.00046200	-0.00015300	0.37045700
F	0.04574500	-1.33858500	-0.12880900
H	0.00005100	0.00016600	1.62536000
F	-1.18296800	0.62965700	-0.12873900
F	1.13696000	0.70899400	-0.12885700

==

^+Hcarb

==

N	-1.09089100	-0.24510300	-0.00007200
N	1.09085500	-0.24509900	-0.00007400
C	-0.67985000	-1.56117400	-0.00005100
C	0.67979500	-1.56117400	-0.00005500
C	-0.00001300	0.52448300	-0.00009100
C	-2.52414000	0.20967700	0.00001600
C	-3.18344300	-0.34236500	-1.26672200
C	-3.18311300	-0.34173000	1.26722200
C	-2.56494500	1.73645800	-0.00038100
C	2.52415100	0.20964500	0.00001800
C	2.56502600	1.73643100	0.00013500
C	3.18330900	-0.34196300	-1.26699000
C	3.18323300	-0.34216300	1.26697000

H	1.37755600	-2.39058100	-0.00000100
H	-1.37757700	-2.39061100	0.00004100
H	-3.17859900	-1.44160200	-1.28742200
H	-4.23215000	-0.01593700	-1.30048100
H	-2.67812900	0.02864900	-2.17016000
H	-2.67773700	0.02998500	2.17033600
H	-4.23189100	-0.01552500	1.30094600
H	-3.17797300	-1.44095400	1.28863000
H	-3.61615100	2.05382700	-0.00045500
H	-2.09627100	2.16348100	0.89893600
H	2.09635400	2.16314500	0.89960000
H	3.61624900	2.05375000	0.00018700
H	2.09642200	2.16328700	-0.89929700
H	4.23205100	-0.01564200	-1.30069700
H	3.17831800	-1.44119000	-1.28812700
H	2.67797100	0.02949000	-2.17023300
H	3.17840300	-1.44139600	1.28786300
H	4.23193300	-0.01571700	1.30085000
H	2.67776000	0.02901400	2.17025100
H	-2.09630200	2.16301000	-0.89994400
H	-0.00004100	1.60618000	-0.00024200

==

$^{+}$Hlut

==

C	-0.00010500	1.85123100	0.00245100
C	1.21049300	1.16032100	-0.00065600
C	1.20652000	-0.22585500	-0.00534900
N	0.00007100	-0.84606400	-0.00730900
H	2.16159500	1.69080100	-0.00149300
H	-0.00016800	2.94238400	0.00498300
H	0.00013200	-1.86489900	-0.01359200
C	-1.21055700	1.16019300	-0.00046700
H	-2.16175300	1.69051300	-0.00114000
C	-1.20638900	-0.22604200	-0.00517400
C	-2.42936300	-1.08455500	0.00421000
H	-2.52953400	-1.59688900	0.97381900
H	-3.32648600	-0.47569700	-0.15436200
H	-2.38314500	-1.84783700	-0.78665300
C	2.42938100	-1.08452000	0.00435900
H	3.32683400	-0.47556900	-0.15177900
H	2.52794500	-1.59872100	0.97318600
H	2.38419900	-1.84627800	-0.78804800

==

$^{+}HMe_3P$

==

P	0.00030800	0.00023900	-0.33687700
C	-0.46943800	-1.65356600	0.21413600
H	0.25379800	-2.38743200	-0.16780700
H	0.00045300	0.00058900	-1.74706100
C	1.66736100	0.42040500	0.21437200
H	-0.47464900	-1.67867700	1.31322800
H	-1.47235700	-1.89504200	-0.16459100
H	2.37845500	-0.32476300	-0.16848600
H	1.93891100	1.41605700	-0.16326000
H	1.69277000	0.42368700	1.31346800
C	-1.19819600	1.23288400	0.21421000
H	-0.90791300	2.22310100	-0.16382500
H	-2.19460600	0.97117700	-0.16815000
H	-1.21783600	1.24938600	1.31334000

==

$^{+}HtBu_3P$

==

H	2.82786800	2.48927200	-0.03666500
H	1.16097700	2.90375100	1.70220900
H	0.95291400	3.63512100	-0.74394100
C	2.38817700	1.49851600	-0.22610500
H	2.87373900	0.79552800	0.46126800
H	2.63778100	1.22639000	-1.26180100
C	0.60021600	1.98638400	1.46714000
H	0.93844400	1.20776500	2.16523600
C	0.36609000	2.72680600	-0.94805000
H	2.12672700	-0.70300400	1.66901500
C	0.87115500	1.61993100	-0.00072400
H	-0.45912900	2.19554900	1.66446000
H	0.52598800	2.46724800	-2.00558600
H	2.93605600	-0.90238700	-0.78907200
H	-0.68696200	2.98489000	-0.80431500
H	1.93451900	-2.46082600	1.69850100
C	1.41825000	-1.51672400	1.46729100
H	0.57383000	-1.42549900	2.16437400
C	2.18150900	-1.67868200	-0.94508400
H	2.66709300	-2.64637400	-0.74818400
H	-2.12124000	2.09116500	0.45983200
P	0.00027900	0.00030300	-0.45199600
C	0.96756400	-1.56417600	-0.00129800
H	1.88227300	-1.67468200	-2.00403700
H	-1.52241600	0.20923900	2.16575000
H	-0.00031400	0.00019600	-1.86679400
C	-2.49008400	1.31947700	-0.22604000
H	-2.37984400	1.67010600	-1.26226400
C	-2.02230600	-0.47525300	1.46652000
C	-1.83892700	-0.05569500	-0.00027100
H	-3.56805800	1.20761400	-0.03556900
H	-1.66924800	-1.49570700	1.66356800
C	0.10363900	-2.81598400	-0.23194300
H	-3.09789100	-0.45413300	1.69841900
H	0.74099900	-3.69346000	-0.04577200
H	-0.74812600	-2.88616800	0.45513600
H	-0.25685600	-2.89184200	-1.26797800
H	-2.39453800	-0.78143100	-2.00609900
C	-2.54573000	-1.04492000	-0.94830100
H	-2.24972300	-2.08711500	-0.79911900
H	-3.62636500	-0.98381800	-0.74945000

==

$^{+}HMes_3P$

==

C	-2.37819400	-3.27158100	0.73687300
C	-3.33192100	-3.08239700	-0.26325600
C	-3.24996000	-1.92564000	-1.04250800
C	-2.26386100	-0.95933300	-0.85090700
C	-1.32199400	-1.17479300	0.18088800
C	-1.37034900	-2.34015600	0.98227400
P	-0.00132800	-0.00044800	0.58162000
C	1.67666700	-0.55940200	0.18671600
C	1.96040400	-1.46435800	-0.86072200
C	3.29023800	-1.83307300	-1.05938200
C	4.33280500	-1.34222000	-0.27049100
C	4.01997200	-0.44162300	0.74878600
C	2.71042600	-0.03574300	0.99945400
C	0.91997300	-2.04399400	-1.78692300
C	5.74962900	-1.78646000	-0.50486100
C	2.45898500	0.94635900	2.11812200
C	-2.26153700	0.24080200	-1.76596300
C	-4.42976000	-4.08318000	-0.49268100
C	-0.37998400	-2.62018200	2.08682700
C	-0.35400500	1.73184000	0.18549200
C	-1.33482600	2.36236600	0.98969600
C	-1.63485300	3.69973700	0.74057600
C	-0.99743400	4.42575500	-0.26744200
C	-0.04169000	3.77168700	-1.04693800
C	0.30034900	2.43360200	-0.85060600
C	-2.07087800	1.64735700	2.09711700
C	1.33080200	1.82663200	-1.77062700
C	-1.32033400	5.87598000	-0.49468400
H	-2.37423200	6.09140300	-0.27115300
H	0.45268900	4.32191900	-1.85052700
H	-0.52975800	-1.94229200	2.94368600
H	-2.50729100	0.69319600	1.76465100
H	1.54458900	2.51468300	-2.59801400
H	-1.40762300	1.43540200	2.95234100
H	-2.39596600	4.19002900	1.35204100
H	2.27745300	1.62273200	-1.24977300
H	-2.41649000	-4.17694800	1.34683900
H	-2.89098800	2.27009000	2.47491300
H	-3.98085300	-1.77202900	-1.83976600
H	1.94020100	0.46877400	2.96599600
H	4.82263300	-0.03553800	1.36834700
H	5.94389900	-1.95653100	-1.57278900
H	0.99308900	0.87768700	-2.21162300
H	3.40847200	1.33709400	2.50441900

H	1.85540400	1.80806700	1.79468100
H	-4.60617600	-4.24184000	-1.56584700
H	-4.19921000	-5.05153300	-0.02968100
H	-5.37236200	-3.71740300	-0.05531400
H	0.66372700	-2.51606000	1.75367300
H	-2.95614000	0.07698200	-2.59946400
H	-2.57481500	1.15632400	-1.24341900
H	-1.27050000	0.43869700	-2.19917500
H	-0.50519300	-3.64286000	2.46296900
H	3.52239500	-2.52686200	-1.87064000
H	6.47145000	-1.04777600	-0.13150400
H	5.94063400	-2.73576700	0.02050100
H	0.24418200	-1.27844100	-2.19384200
H	1.40796200	-2.53622200	-2.63758000
H	-0.00579800	-0.00042000	1.98727400
H	0.29221200	-2.79158700	-1.28014900
H	-0.70554000	6.50885100	0.16521100
H	-1.11414600	6.17700100	-1.53055200

==

$H_3P–BF_3$

==

P	-1.95427000	-0.00085600	0.00169800
B	1.12698700	0.00034100	-0.00055100
F	1.17746500	1.12606200	0.69799100
F	1.18129200	-1.16755900	0.62475100
F	1.17309600	0.04271800	-1.32481400
H	-2.70797300	-0.10307600	1.20739900
H	-2.69908200	1.09881400	-0.51624500
H	-2.70051200	-0.99558500	-0.69521800

==

$H_3P\cdots H_2\cdots BF_3$

==

P	-1.90800400	-0.00365100	-0.13460200
B	1.04412500	0.01359900	-0.24256300
H	-0.78677600	0.09943700	-1.10030200
H	0.54215100	-0.00548600	-1.43351000
H	-1.61815300	0.63796400	1.07859000
H	-3.12028900	0.58438900	-0.57253200
H	-2.27491700	-1.32944700	0.14893300
F	0.77952200	1.27815400	0.31548600
F	2.36840000	-0.32949000	-0.27263600
F	0.25845800	-0.94867400	0.52500100

==

$H_3PH\cdots HBF_3$

==

P	1.62794200	0.00887700	-0.16652900
B	-1.35439300	-0.01979900	0.26665100
H	0.66460100	0.89566300	-0.70704200
H	-2.38570200	-0.01563800	0.90393600
H	1.22648500	-1.33108800	-0.17799700
H	2.76683100	0.03632900	-1.01643400
H	2.15398400	0.40358000	1.06996700
F	-1.14790400	1.15934600	-0.52452100
F	-1.13082900	-1.14233500	-0.55921500
F	-0.17386300	-0.01956800	1.20509700

==
